# Mechanistic Investigation of *tert*-Butanol’s Impact on Biopharmaceutical Formulations: When Experiments Meet Molecular Dynamics

**DOI:** 10.1021/acs.molpharmaceut.3c00125

**Published:** 2023-07-12

**Authors:** Marcello Rospiccio, Paola Casucci, Andrea Arsiccio, Claudia Udrescu, Roberto Pisano

**Affiliations:** Molecular Engineering Laboratory, Department of Applied Science and Technology, Politecnico di Torino, Torino 10129, Italy

**Keywords:** *tert*-butyl alcohol, protein stability, freeze-drying, cosolvent formulations, molecular dynamics, cyclodextrins

## Abstract

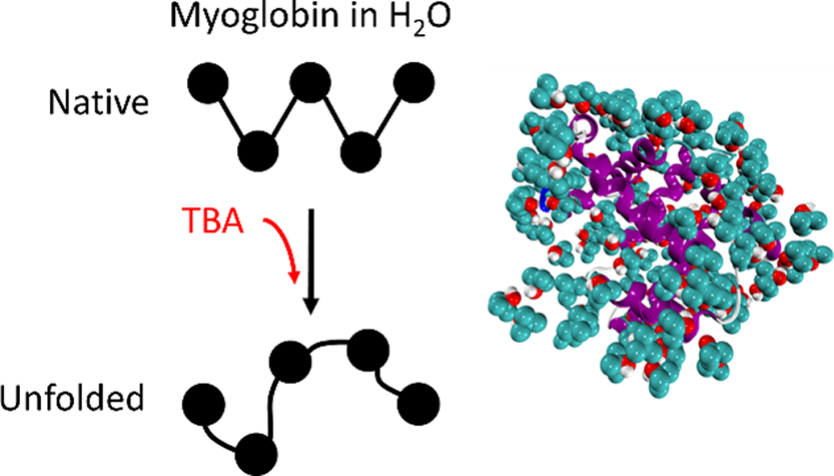

The use of *tert*-butyl alcohol for the lyophilization of pharmaceuticals has seen an uptick over the past years. Its advantages include increased solubility of hydrophobic drugs, enhanced product stability, shorter reconstitution time, and decreased processing time. While the mechanisms of protein stabilization exerted by cryo- and lyo-protectants are well known when water is the solvent of choice, little is known for organic solvents. This work investigates the interactions between two model proteins, namely, lactate dehydrogenase and myoglobin, and various excipients (mannitol, sucrose, 2-hydroxypropyl-β-cyclodextrin and Tween 80) in the presence of *tert*-butyl alcohol. We thermally characterized mixtures of these components by differential scanning calorimetry and freeze-drying microscopy. We also spectroscopically evaluated the protein recovery after freezing and freeze-drying. We additionally performed molecular dynamics simulations to elucidate the interactions in ternary mixtures of the herein-investigated excipients, *tert*-butyl alcohol and the proteins. Both experiments and simulations revealed that *tert*-butyl alcohol had a detrimental impact on the recovery of the two investigated proteins, and no combination of excipients yielded a satisfactory recovery when the organic solvent was present within the formulation. Simulations suggested that the denaturing effect of *tert*-butyl alcohol was related to its propensity to accumulate in the proximity of the peptide surface, especially near positively charged residues.

## Introduction

In recent years, the interest in biopharmaceutical products has increased^1^ thanks to their superior biological activity and selectivity. However, besides their appealing features, these drug products pose new challenges in manufacturing and formulation design.^2,3^ In fact, biopharmaceutical products are frequently made up of proteins, which are often susceptible to various environmental stressors. Sources of stress may be encountered throughout the production and during the subsequent storage of the drug product. For this reason, it is necessary to carefully evaluate the impact of all possible harmful conditions throughout the whole life cycle of the biopharmaceutical product. The physical, chemical and biological stability are specific to each product, but some general guidelines can be provided; the most common stressors are low and high temperatures, pH shifts, destabilizing solutes or interfaces, dehydration and undesired chemical reactions, possibly mediated by microbial activity.^2,3^ These factors may cause protein denaturation and/or aggregation,^4^ potentially leading to loss of biological activity and even harmful immune responses.^5^

Since the solvent mediates many destabilizing phenomena, it is often necessary to drastically reduce its content in the final biopharmaceutical product and, hence, ensure its stability and preserve its biological activity over time. However, proteins are thermolabile substances and, thus, some of the techniques used to remove solvents, such as evaporation, may not be viable as the temperatures achieved during the process could lead to denaturation. In the pharmaceutical industry, this problem is often overcome by either freezing or freeze-drying, yielding a product in a solid form. However, both freezing and freeze-drying may introduce other sources of stress, potentially harmful to proteins, and hence require the addition of excipients to preserve the therapeutic features of the drug products^3^ throughout the manufacturing process and during their storage. Many different chemical compounds can be used as excipients, for example, sugars, polyols, surfactants, amino acids, salts/buffers and polymers. While the interactions of these excipients with proteins have been the subject of extensive investigation in presence of water as solvent, little is known in the case of organic solvents or mixtures of water and organic solvents.

**Figure 1:**
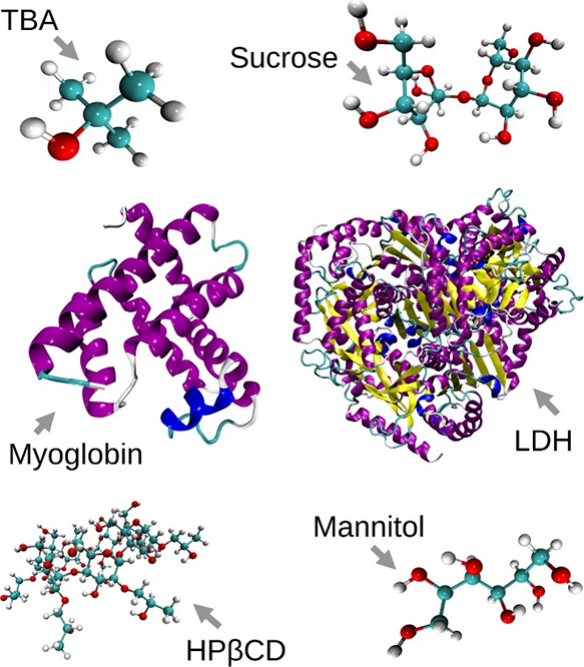
Snapshots of the simulated molecules realized with Visual Molecular Dynamics 1.9.3 (VMD).^23^

Choosing the most appropriate solvent for a drug product is a demanding task,^6^ especially considering the possible implications on fragile compounds such as proteins. Mixtures of organic solvents and water are not uncommon in the lyophilization of drug products.^7^ One of the most common cosolvents is *tert*-butyl alcohol (TBA), whose structure is shown in Figure 1. TBA has a relatively high freezing point,^8^ and it is often used to speed up the freeze-drying process^7−11^ or improve the solubility of hydrophobic substances.^12^ However, the use of this solvent requires attention and appropriate handling because of its high vapor pressure and flammability.^9^

Over the years, there have been a few investigations focusing on the use of TBA and TBA/water mixtures to solubilize proteins, revealing that this cosolvent generally had a detrimental effect on thermal stability and on the biological activity.^13,14^ Yong et al.^15^ and Sonje et al.^16^ have shown that the presence of other additives/excipients, in combination with TBA, may be needed to ensure protein stability throughout common processes, such as freezing or freeze-drying. The number of protein molecules whose stability in TBA/water has been assessed is, however, quite limited. Moreover, no general guideline exists about the type or dosages of excipients to guarantee an acceptable protein recovery in presence of TBA, and the mechanism of TBA-induced protein destabilization is not unambiguous. For these reasons, further research in this area is required, especially to identify any type of synergistic or antagonistic interplay between TBA and common pharmaceutical excipients.

This work aims at clarifying the role played by TBA in the excipient-protein and solvent-protein interactions during freezing and drying. More specifically, formulations containing lactate dehydrogenase (LDH) and myoglobin (Mb) were investigated to assess any potential onset of either surface-induced or cold denaturation.^17,18^ LDH was chosen as it is an excellent candidate, since its behavior upon freezing and freeze-drying is well documented in literature, in combination with many types of excipients as well. On the other hand, Mb was chosen as some research has been conducted on the effect of cosolvents (among which TBA) on its thermal stability,^13^ although without characterization of their impact upon freezing and/or freeze-drying. Additionally, the combination of these two proteins for stability studies regarding freezing and freeze-drying has already been reported in literature.^17,18^ We studied various formulations for LDH and Mb, containing combinations of citrate buffer, mannitol, sucrose, 2-hydroxypropyl-β-cyclodextrin (HPβCD), Tween 80, and different water-TBA mixtures. First, all the formulations were thermally characterized by low-temperature differential scanning calorimetry (DSC) and freeze-drying microscopy (FDM); then, the protein recovery was spectroscopically evaluated after freeze/thawing and freeze-drying. Mannitol formulations were characterized by X-ray diffractometry (XRD) to determine the crystalline forms of this excipient. Experimental data were supported by molecular dynamics^19^ (MD) simulations. MD is a valuable tool in the formulation field,^20,21^ providing detailed information at a molecular level about the mechanisms of interactions between the various ingredients of a formulation.^22^ Here, MD simulations aimed at understanding the mechanisms of interactions between TBA, excipients and the two model proteins.

## Methods

### Materials

Two model proteins were selected for the experimental investigation, myoglobin from equine heart (Merck, Milano, Italy) and lactate dehydrogenase from rabbit muscle (Roche, Monza, Italy). Mb (pI 6.8–7.4) was used as supplied, and dissolved in 10 mM sodium citrate buffer at pH 3.7.^17,18^ LDH (pI 7.1) was dialyzed against 10 mM sodium citrate buffer at pH 6.5.^17,18^ Dialysis was performed at 4 °C and the buffer was changed three times (the first two times every 3 h, whereas the third dialysis step was carried out overnight). The concentration of LDH after dialysis was determined using UV/vis spectroscopy (Multiscan Sky spectrophotometer, ThermoScientific, Milano, Italy). The peak at 280 nm was monitored and an extinction coefficient of 1.44 mL/(mg cm) was used for the calculations.

Citrate buffer was selected for Mb and LDH because it does not undergo selective precipitation during freeze–thawing experiments and, thus, should guarantee an accurate control of pH.^24^ Various formulations were designed using different excipients and varying the concentration of TBA, as shown in Table 1. Some of these formulations also contained Tween 80 at 0.01% w/v concentration. In all the formulations, the concentrations of Mb and LDH were 0.1 mg/ml^18^ and 5 μg/ml,^25^ respectively. The excipients, TBA and Tween 80 were obtained from Sigma-Aldrich (Milano, Italy) and used as supplied.

**Table 1:** List of all the Formulations Studied in this Work^a^

code	formulation
B	
M	5% w/w mannitol
S	5% w/w sucrose
H	5% w/w HPβCD
B*	0.01% w/v Tween 80
M*	5% w/w Mannitol + 0.01% w/v Tween 80
S*	5% w/w Sucrose + 0.01% w/v Tween 80
H*	5% w/w HPβCD + 0.01% w/v Tween 80
Bo	20% w/w TBA
Mo	5% w/w Mannitol + 20% w/w TBA
So	5% w/w Sucrose + 20% w/w TBA
Ho	5% w/w HPβCD + 20% w/w TBA
Bo*	20% w/w TBA + 0.01% w/v Tween 80
Mo*	5% w/w Mannitol + 20% w/w TBA + 0.01% w/v Tween 80
So*	5% w/w Sucrose + 20% w/w TBA + 0.01% w/v Tween 80
Ho*	5% w/w HPβCD + 20% w/w TBA + 0.01% w/v Tween 80
Bo5	5% w/w TBA
Bo10	10% w/w TBA
Bo30	30% w/w TBA

a They were prepared in 10 mM citrate buffer at pH 3.7 for Mb and at pH 6.5 for LDH, respectively. Formulation B corresponds to the buffer only.

### Differential Scanning Calorimetry (DSC)

The DSC analyses were carried out using a differential scanning calorimeter (DSC type Q200, TA Instruments, New Castle, DE, USA) that uses a refrigerated cooling system and nitrogen for cell purge (at 50 mL/min). A small amount (between 25 and 35 mg) of the selected (protein-free) formulation was loaded into an aluminum pan, and hermetically sealed. The sample was then frozen at −80 °C, using a cooling rate of 1 °C min^–1^ and, then, heated at 1 °C min^–1^ up to room temperature. The DSC analyses were performed on the formulations at pH 3.7 and pH 6.5 to ensure that the pH does not affect the observed thermal transitions.

### Freeze Drying Microscopy (FDM)

The FDM analyses were carried out using a freeze-drying microscope (microscope: BX51, Olympus Europa, Hamburg, Germany; temperature controller: PE95-T95, Linkam, Scientific Instruments, Tadworth, Surrey, UK). A small aliquot of the protein-free solutions was cooled down to −60 °C at 5 °C min^–1^ and atmospheric pressure, then heated at 10 Pa using different heating rates (2 °C min^–1^ up to −30 °C, 1 °C min^–1^ up to −10 °C and 5 °C min^–1^ up to 20 °C). The analyses were performed at pH 3.7 and pH 6.5.

### Freezing and Freeze-Drying Protocols

Freezing experiments were performed at both high (in liquid nitrogen) and low (at 0.3 °C min^–1^) cooling rates, using 1 mL of solution in 4R vials (Bertolini S.p.A., Candiolo, Italy), which were sealed using igloo-type stoppers (West Pharmaceutical Services, Milano, Italy).

In the case of fast freezing, vials were immersed in liquid nitrogen for 2 min and then thawed at room temperature. This procedure was repeated three times for Mb and only once for LDH.

In the case of slow freezing, vials were loaded at room temperature in a laboratory-scale freeze-dryer (LyoBeta 25, Azbil Telstar, Terrassa, Spain). Then, the shelf temperature was reduced to −45 °C at 0.3 °C min^–1^ and held at −45 °C for 30 min to ensure the complete freezing of the solution. Once frozen, the vials were heated up to 20 °C at 0.3 °C min^–1^ and maintained at that temperature for 30 min to complete their thawing. We performed three freeze–thaw cycles for Mb and one for LDH.

The freeze-drying runs were carried out in a laboratory-scale freeze-dryer using the protocol shown in Table 2. The primary drying endpoint was detected by comparing the pressure measured by a capacitive sensor (Baratron, type 626A, MKS Instruments, Andover, MA, USA) and a Pirani gauge (type PSG-101-S, Inficon, Bad Ragaz, Switzerland).^26,27^

**Table 2:** The Freeze-Drying Protocol Employed for Mb and LDH Formulations*

phase	description	temperature, °C	time, min	pressure, Pa
1	loading	20		
2	cooling	–45	217	
3	holding	–45	120	
4	vacuum	–45		10
4	primary drying – ramp	–25	40	10
5	primary drying – holding	–25	*	10
6	secondary drying – ramp	20	240	10
7	secondary drying – holding	20	300	10

* as indicated by the comparative Baratron/Pirani pressure measurement.^26^

### Characterization of Mannitol-Based Formulations – X-ray Diffractometry

For mannitol formulations (M, M*, Mo, Mo*, see Table 1 for further details), X-ray diffractometry (XRD, X-pert Powder type, PANalytical, Almelo, Netherlands) was used to identify the polymorphic state of the lyophilized samples. The diffraction profiles were collected in the 2θ range between 5° and 90°, with an acquisition step of 0.018° and a time per step of 10 s using a solid state PIXcel-1D detector with 255 active channels.

Samples’ structure was studied by X-ray diffraction (XRD) analysis, carried out using a high-resolution Philips X’pert MPD powder diffractometer (The Netherlands), equipped with Cu Kα radiation (*V* = 40 kV, *I* = 30 mA) and a curved graphite secondary monochromator. The X-ray diffraction patterns acquired were compared to those of the reference materials (α, β and δ-anhydrous mannitol).

### Measurement of the Protein Recovery

After freeze/thawing and freeze-drying, the Mb solutions were centrifuged at 13,000 rpm for 5 min (Heraeus Megafuge 8 Centrifuge Series, Thermo Fisher Scientific, Milano, Italy). After centrifugation, the percentage of aggregates was calculated as the decrease in absorbance at 280 and 410 nm with respect to the initial value, whose complementary yields the corresponding recovery. Optical density (OD) was measured using a Multiscan Sky spectrophotometer (ThermoScientific, Milano, Italy) against a solvent-matched reference.^17^ The peak centered at 280 nm in the Mb absorbance spectrum is due to aromatic amino acids (primarily tryptophan and tyrosine) and the heme iron. The other absorption peak centered at 410 nm is entirely owing to the heme and is referred to as the Soret band.^28^

The enzymatic activity of LDH after freeze/thawing and freeze-drying was calculated from the increase in absorbance at 450 nm due to the reduction of NAD^+^ to NADH (Lactate Dehydrogenase Activity Assay Kit, Sigma-Aldrich, Milano, Italy). A standard curve built with 1.25 mM NADH standard was used to calculate the amount of NADH generated in each well.^17^

### Statistical Analysis

At least three samples were collected for each combination of formulation/stress protocol, and each sample was analyzed at least in duplicate. The presence of statistically relevant differences was identified by application of the analysis of variance (ANOVA) technique, followed by the honestly significant difference (HSD) or Tukey test. The software Minitab 17 was employed for this purpose.

### Simulation Details

Molecular Dynamics(MD) simulations are here used to describe the complex molecular interactions between proteins, excipients and TBA molecules. This study was limited to Mb formulations, as this protein is much smaller than LDH (153 vs 571 amino acid residues) and, thus, more amenable to be analyzed by MD simulations.

At first, we developed and validated a topology file for TBA molecules; then, we investigated the interactions between Mb and TBA in the presence of various excipients. This analysis investigated the behavior both in the bulk solution and at the air–water interface. A detailed description of the simulations set-up is given in Table 3. Specifically, all the myoglobin-containing simulations were carried out using a higher protein concentration than the experimental one, in order to limit the size of the simulation box. All the simulations were performed using Gromacs 2020.1.^29^

**Table 3:**
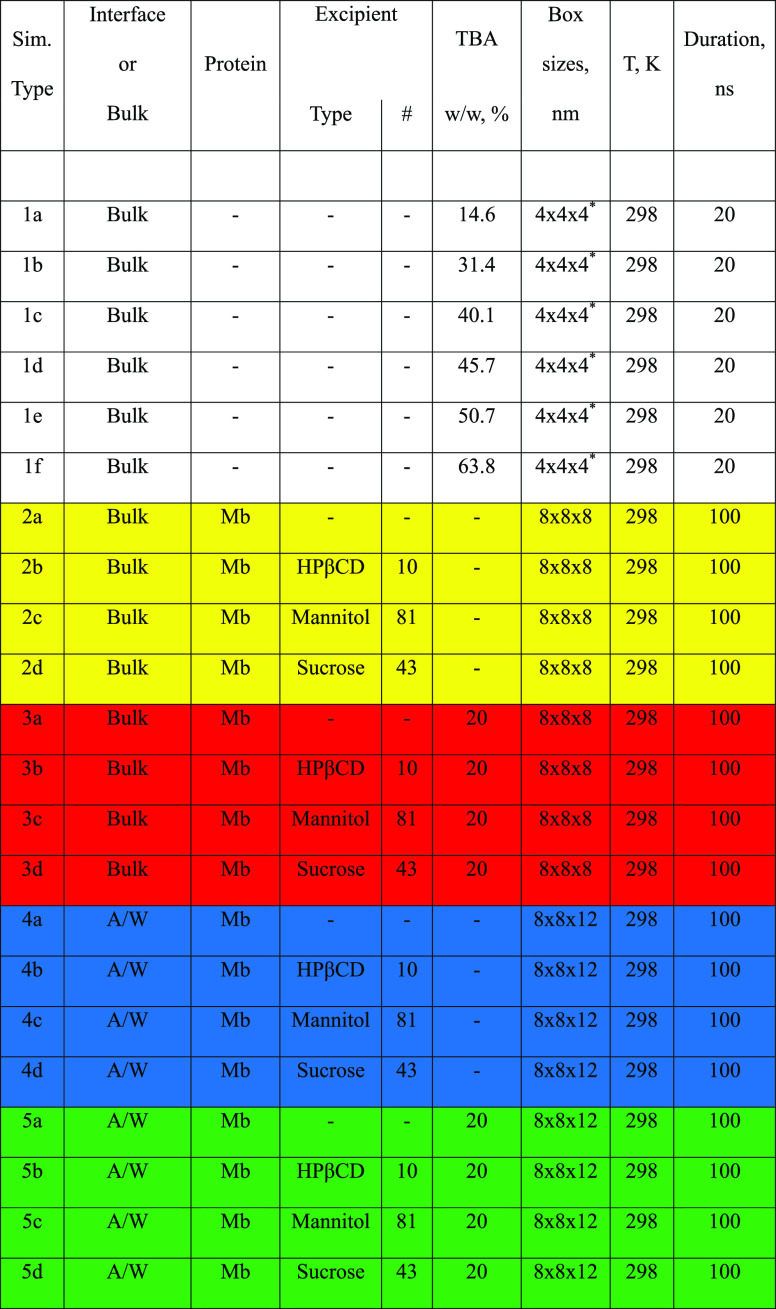
Summary of the MD Simulations Performed in this Work

A/W: air–water. Color code: white, TBA/water mixtures; yellow, myoglobin formulations in aqueous bulk; red, myoglobin formulations in aqueous bulk with TBA; blue, myoglobin formulations at the A/W interface; green, myoglobin formulations at the A/W interface with TBA.

* These simulations were also run in identical systems of size 8 × 8 × 8 nm.

### TBA Force Field

As first attempt, TBA was described with the GROMOS 53a6-compatible^30,31^ parameterization developed by Lee and van der Vegt,^32^ which was validated upon the Kirkwood-Buff integrals^33^ (KBIs) determined experimentally by Nishikawa et al.^34^ Subsequently, we developed a modified parameterization (TBAff) for the nonbonded interactions of TBA oxygen atom with its carbon atoms, in order to improve the reproducibility of the physical behavior of TBA-water mixtures, as the microstructure of cosolvent mixtures has been observed to affect the biological activity of Mb.^13^ This parameterization was validated and optimized upon the experimental KBIs published in Nishikawa et al.,^34^ jointly with two different water force field/combination rule sets, i.e., SPCE^35^/combination rule 1 or CHARMM TIP3P^36^/combination rule 2 (Table 4). All the simulations in Table 3 were performed for both the two water force field/combination rule sets.

**Table 4:** TBAff Parameterizations for TBA’s Non-bonded Interactions

	SPCE/combination rule 1		CHARMM TIP3P/combination rule 2	
	C^6^, nm^6^ kJ/mol	C^12^, nm^12^ kJ/mol	σ, nm	ε, kJ/mol
atoms non-bonded parameters for TBA				
hydrogen	0	0	0	0
oxygen	2.261954E–03	1.505529E–06	0.2955	0.8496
carbon	2.397081E–03	2.053489E–04	0.6639	0.007
methyl	9.613802E–03	2.664624E–05	0.3748	0.8672
oxygen non-bonded parameters for TBA				
carbon–oxygen	2.1344443E–03	1.950104E–07	0.3846	0.1123
methyl–oxygen	5.321969E–04	1.502845E–06	0.3271	0.4492

The TBAff parameterization was validated for various water-TBA mixtures, referring to the different TBA concentrations already characterized by Nishikawa et al.,^34^ i.e., 14.6, 31.4, 40.1, 45.7, 50.7 and 63.8% by weight (corresponding to 4, 10, 14, 17, 20 and 30% in terms of mole fraction, respectively) (sims. #1a–1f). Independently of the case study, the simulation box was a 4-nm-edge cube and the system was energy-minimized using the steepest descent algorithm. Then, it was equilibrated for 1 ns in the NPT ensemble using the Berendsen thermostat^37^ at 298 K (1 ps relaxation time) coupled with the Berendsen barostat^37^ at 1 bar (1 ps relaxation time), with a 2 fs time-step. The cut-off radius used for both non-bonded Lennard-Jones and Coulomb potentials was 1.2 nm. Periodic boundary conditions were used. The PME^38^ approach was used to evaluate the long-range electrostatics interactions. The Lincs^39^ algorithm was used to constrain all bonds, while the SETTLE^40^ algorithm was used to keep water molecules rigid. The mixtures were then simulated for 20 ns in the same conditions, using the Nosé-Hoover thermostat^41−43^ (1 ps relaxation time) and the Parrinello-Rahman barostat^44^ (3 ps relaxation time). The trajectories were then used to compute the radial distribution functions (rdf from here on), in order to calculate the KBIs:
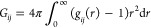
1where *G_ij_* represents the KBI, *g_ij_* is the rdf, i.e., the function that describes the density of the species *j* as a function of the distance *r* from the species *i*. In other terms, a value of *G_ij_* > 1 indicates accumulation of *j* with respect to *i*, while a value of *G_ij_* < 1 suggests exclusion. Since computational evaluations of KBIs may suffer from finite size effects, a corrected form of rdf was adopted.^45^ For the same reason, we also decided to further validate the TBAff parameterization enlarging the simulation box, i.e., using an 8-nm-edge cube (sims. #1a–1f).

### Simulation of Myoglobin Formulations

The molecular structure of myoglobin was obtained from the RCSB PDB data bank,^46^ PDB code 1WLA.^47^ The protein conformation at pH 3.7 was then obtained from the H++ server,^48^ resulting in a charge equal to +10, which was balanced by the addition of a corresponding number of Cl^–^ counterions (see the Myoglobin Setup section of the Supporting Information for further details). For the simulations of myoglobin in bulk, we used the same methodology and parameters described in the previous section (type #1 simulations) and a simulation time of 100 ns. The simulation box was a 8-nm-edge cube containing one Mb molecule and a number of molecules of excipients and TBA equivalent to a weight concentration of 5 and 20%, respectively. The codes corresponding to each simulation type are reported in Table 3. It should be noted that the protein concentration in all simulations was higher than the experimental one: such compromise was necessary in order to obtain a simulation box of reasonable size.

In the case of myoglobin at the air–water interface, simulations were equilibrated for 1 ns in the NVT ensemble with the V-rescale^49^ thermostat at 298 K (1 ps relaxation time). The simulation time was 100 ns, while the simulation box was composed of a 8-nm-edge cube filled with the protein formulation, plus an extra 4-nm-long vacuum space along the *z* axis. Overall, the simulation box was 8 × 8 × 12 nm.

All the simulations type #2–5 were repeated twice, using both a CHARMM36m^50^ and a GROMOS 54a7^51−53^ description for Mb, in combination with CHARMM TIP3P^36^ and SPCE^35^ water, respectively. The SPCE/combination rule 1 parameterization of TBA previously developed (Table 4) was used in combination with GROMOS 54a7, while the CHARMM TIP3P/combination rule 2 description of TBA was combined with CHARMM36m for myoglobin. This choice gave us the opportunity to compare the results obtained from different parameterizations of the same system, increasing the overall reliability of the simulations. In all the simulations, mannitol, sucrose and HPβCD were modeled with the ADD force field.^54,55^

The average radius of gyration and hydrophobic fraction of solvent accessible surface area of myoglobin were computed using the built-in Gromacs commands (*gmx gyrate* and *gmx sasa*(56)). All the analyses have been performed on the last 80 ns of the simulated trajectories.

The β-parameter^20,57^ profile was computed starting from the rdf for all those systems containing excipients and/or TBA, i.e., all the simulations type #2–5. The β-parameter profile is defined as,
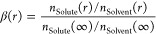
2where *n*_solute_(*r*) and *n*_solvent_(*r*) represent, respectively, the number of solute (excipients/TBA) or solvent (water) molecules at distance *r* from the protein’s surface, while *n*_solute_(*∞*) and *n*_solvent_(*∞*) refer to the number of solute and solvent molecules in the system. This parameter is used to describe the spatial profile of the degree of preferential exclusion of solutes from the peptide surface; a value of β(*r*) > 1 indicates preferential inclusion, while a value <1 indicates preferential exclusion.

In order to better understand the mechanisms of interactions between TBA and myoglobin, we identified the peptide residues that, on average, were within a spherical neighborhood of 0.3 nm from the center of mass of the TBA molecules.^57^ Lastly, the dimensionless density profiles were computed for myoglobin, excipients and TBA at the A/W interface to assess the impact of interfacial interactions, if any.

## Results and Discussion

### Thermal Characterization

All the formulations listed in Table 1 were thermally characterized by DSC and FDM. As the protein concentration was low (0.1 mg/mL for Mb and 5 μg/mL for LDH), we can assume that the thermal behavior of the formulations is not affected by the presence of the protein and, thus, all the thermal analyses were carried out using placebo formulations. Table 5 shows the results of this analysis in terms of the glass transition temperature (*T*′_g_), crystallization temperature (*T*_cr_), and eutectic melting temperature (*T*_eu_) as measured by DSC, and the collapse temperature (*T*_c_) as determined by FDM.

**Table 5:** Results of the DSC and FDM Analyses^a^^,^^b^

formulation	pH 3.7				pH 6.5			
	*T*′_g_, °C	*T*_cr_, °C	*T*_eu_, °C	*T*_c_, °C	*T*′_g_, °C	*T*_cr_, °C	*T*_eu_, °C	*T*_c_, °C
M	–31.1	–34.5 (*C*)			–35.8	–37.8 (*C*)		
		–23.8 (*H*)				–29.7 (*H*)		
S	–33.5			–31.9	–36.9			–32.2
H	–17.3			–14.6	–17.7			–14.9
M*	–31.3	–34.2 (*C*)			–35.9	–37.4 (*C*)		
		–26.7 (*H*)				–28.4 (*H*)		
S*	–33.4			–32	–37.0	-		–32.1
H*	–17.8			–14.2	X			
Bo		–18.9	–7.8		X			
Mo	–32.4	–35.4 (*C*)	–7.9	–28.2	–35.6	–37.9 (*C*)	–9.3	–28.2
		–26.3 (*H*)				–28.7 (*H*)		
		–20.2 (*H*)						
So	–41.5	–23.3 (*H*)	–8.3	–33.1	–38.2	–27.8 (*H*)	–10.3	–32.1
Ho	–33.5	–16.5	–7.8	–18.3	X			
	–23.6							
Bo*			–12.6		X			
			–7.9					
Mo*	–32.0	–34.5 (*C*)	–11.8	–29.2	–28.6	–37.9 (*C*)	–13.6	–26.3
	–26.4	–22.6 (*H*)	–8.3			–28.6 (*H*)	–9.7	
So*	–39.1		–14.9	–33.1	–38.3		–10.1	–32.1
			–8.7					
Ho*	–29.5		–11.1	–18.2	X			
	–23.4		–8.4					
Bo5			–9.8		X			
			–8.6					
			0.9					
Bo10			–10.1		X			
			–8.3					
			–2.5					
Bo30		–11.6	–8.0		X			
			–5.9					

a *T*′_g_: glass transition temperature of the maximally freeze-concentrated solution, *T*_cr_: crystallization temperature, *T*_eu_: eutectic melting temperature, *T*_c_: collapse temperature as determined by FDM, *C*: cooling, *H*: heating.

b The table entries marked with ‘X’ indicate that measurements were not performed at that pH for the formulation under exam.

The formulations were analyzed at both pH 3.7 and pH 6.5, and we did not observe any significant difference in the thermal behavior. For example, in the case of the H formulation, the glass transition temperature of the maximally freeze-concentrated solution *T*′_g_ was about −17 °C, while the collapse temperature was about −15 °C. It follows that HPβCD remained amorphous during freezing.

For the mannitol solution (M), we identified a crystallization event during cooling (at −34.5 °C for pH 3.7 and at −37.8 °C for pH 6.5). However, the crystallization was not complete as we observed a glass transition event at −31.1 °C (at pH 3.7) and at −35.8 °C (at pH 6.5),^58^ followed by a second crystallization event during the heating ramp at −23.8 °C (at pH 3.7) and at −29.7 °C (at pH 6.5), respectively.

While mannitol is a crystalline excipient, sucrose generally remains amorphous. Indeed, for formulation S at pH 3.7 we observed a glass transition temperature at −33.5 °C (in line with the literature data^59^) and its collapse at −31.9 °C, while at pH 6.5 the thermal events were found at −36.9 °C (*T*′_g_) and – 32.2 °C (*T*_c_), respectively.

The addition of 0.01% w/v Tween 80 to mannitol, sucrose and HPbCD formulations (M*, S*, and H*) did not alter the thermal behavior of the excipients. Therefore, the surfactant seemed not to have any meaningful effect at this low concentration.

We then analyzed the thermal behavior of TBA/citrate buffer solutions containing 5%, 10%, 20%, and 30% by weight of TBA (Bo5, Bo10, Bo, and Bo30 in Table 3). For the Bo5 formulation, we observed three endothermic peaks corresponding to the dihydrate-ice eutectic (−9.8 °C), heptahydrate-ice eutectic (−8.6 °C), and ice melting (0.9 °C), in agreement with previous experimental observations.^60^ Bo10 resulted in a similar DSC thermogram. By contrast, the Bo formulation was close to its eutectic composition^61^ (i.e., 22.5% w/w^7^), hence it showed the TBA heptahydrate crystallization at −18.9 °C and the TBA heptahydrate-ice eutectic melting at −7.8 °C.^16^ Lastly, Bo30 showed a crystallization event at −11.6 °C due to the TBA heptahydrate, and two melting points at −8.0 and −5.9 °C related to the TBA dihydrate-ice and TBA heptahydrate-ice eutectic, respectively.

The Mo formulation contained mannitol in a TBA/buffer mixture close to its eutectic concentration. During cooling, mannitol partly crystallized at −35.4 °C (pH 3.7) and −37.9 °C (pH 6.5), while, during the heating phase, we observed a glass transition at −32.4 °C (pH 3.7)/–35.6 °C (pH 6.5). Furthermore, two exothermic events were observed at pH 3.7, at −26.3 and −20.2 °C, and one exothermic event at pH 6.5, at −28.7 °C. The last two peaks could hardly be attributed, due to the superposition of both mannitol and TBA thermal transitions. Finally, melting of the TBA heptahydrate-ice eutectic was detected at −7.9 °C (pH 3.7) and −9.3 °C (pH 6.5).

The addition of TBA to the two amorphous excipients, sucrose and HPβCD (i.e., So and Ho formulations), lowered their glass transition temperatures. *T*′_g_ was −41.5 °C (pH 3.7) and −38.2 °C (pH 6.5) for So, while the Ho formulation showed two glass transition events at −33.5 and −23.6 °C (only pH 3.7 was analyzed in this case). The addition of TBA translated into a marked decrease in the collapse temperature for the Ho formulation (from −14.6 °C for H to −18.3 °C for Ho), while the *T*_c_ of sucrose was not dramatically affected. The DSC thermogram showed the TBA heptahydrate’s crystallization at −23.3 °C (pH 3.7)/–27.8 °C (pH 6.5) for So, at −16.5 °C for Ho and the TBA heptahydrate-ice eutectic melting at −8.3 °C (pH 3.7)/–10.3 °C (pH 6.5) for So and – 7.8 °C for Ho, respectively.

We also observed that Tween 80 (see Bo*, Mo*, So*, and Ho* formulations) led to the disappearance of the crystallization peak for the TBA heptahydrate during the heating phase of the DSC, suggesting that TBA heptahydrate may already form contextually to ice during the cooling phase. Furthermore, there were two eutectic transitions: the melting of the TBA dihydrate at around −12 °C and that of the TBA heptahydrate at around −8 °C. For Mo*, mannitol crystallized during both cooling and heating, but a second glass transition appeared at −26.4 °C, at pH 3.7. Lastly, the addition of TBA lowered the glass transition temperatures of the amorphous excipients (So* and Ho* with respect to S* and H*) and their collapse temperature. Furthermore, XRD characterization of mannitol formulations showed that the addition of TBA promoted the formation of δ-mannitol, as reported in literature.^16,62^ Further details are available in the Supporting Information.

### Protein Recovery

**Figure 2:**
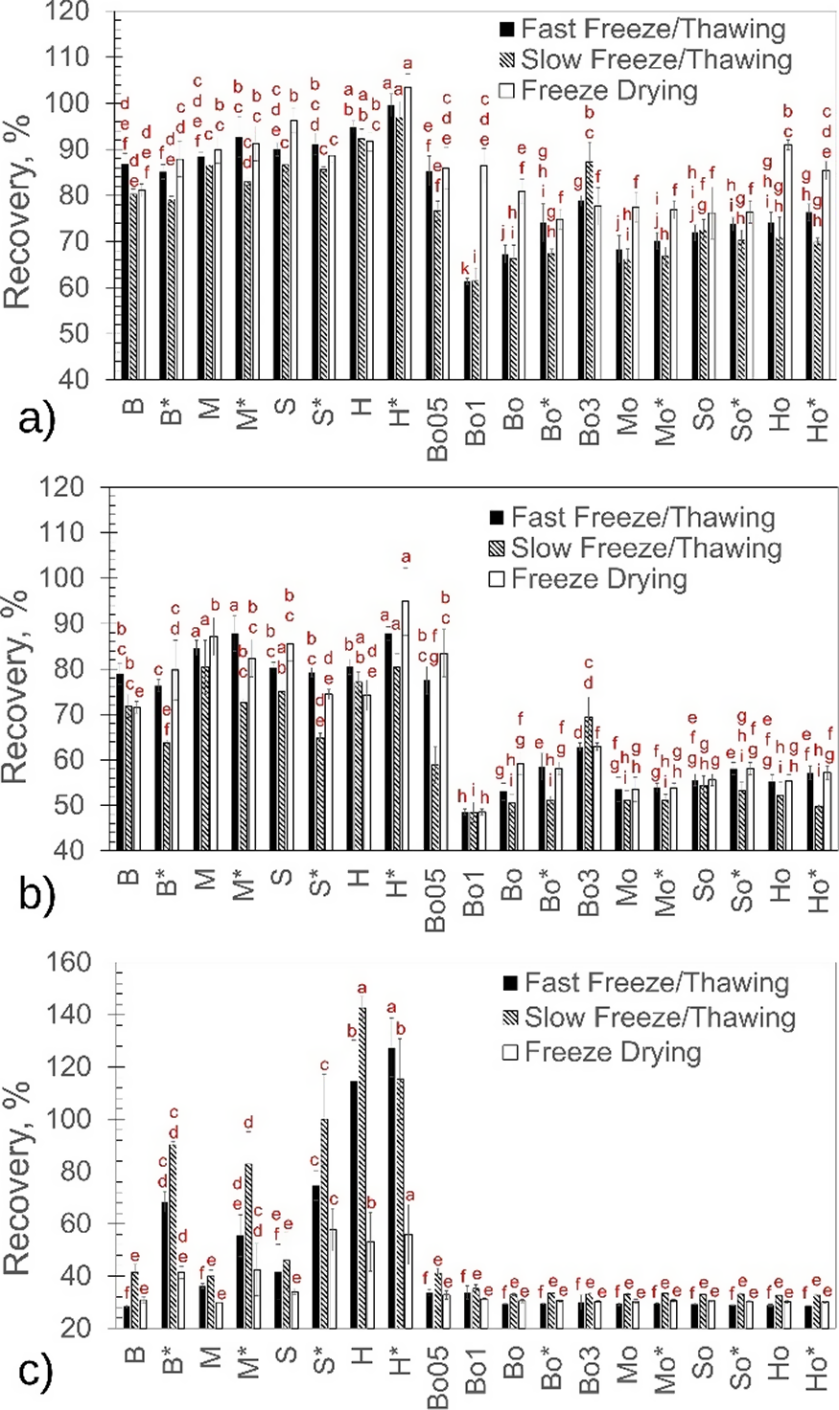
Recovery of protein after fast (black columns) and slow (dashed columns) freeze/thawing or freeze-drying (white columns). According to ANOVA (*p*-value < 0.05), columns with different letters are statistically different. Myoglobin recovery as measured by OD at 280 nm (a) and at 410 nm (b). Lactate dehydrogenase recovery as measured by enzymatic activity (c).

The recovery of myoglobin and lactate dehydrogenase was evaluated after freeze/thawing and freeze-drying for all the formulations described in Table 1. In the case of myoglobin, the absorbance of the solution was measured at both 280 and 410 nm. The OD at 280 nm estimates the aggregation of Mb, while a decrease in the absorbance at 410 nm results from protein aggregation and denaturation.^17^ As shown in Figure 2a,b, the Mb recovery was higher in the case of freezing in liquid nitrogen than that observed for the slow freezing. Mb at pH 3.7 is extremely sensitive to cold denaturation and, hence, its denaturation and aggregation become more pronounced as the residence time at low temperature increases.

Similarly, Figure 2b shows that the OD at 410 nm drastically decreased after freeze-drying and, thus, freeze-drying was detrimental to the Mb recovery, especially in terms of protein denaturation. Overall, the addition of TBA further promoted the Mb aggregation (Figure 2a) and denaturation (Figure 2b). The addition of excipients was beneficial against Mb aggregation and denaturation only in the absence of TBA, and the combination of cyclodextrin and Tween 80 proved to be particularly effective. Nonetheless, none of the tested excipients could mitigate the denaturing effect of TBA.

Interestingly, mannitol protected myoglobin comparably or even better than sucrose, although its crystalline nature. This result confirms that myoglobin denaturation was promoted by cold unfolding rather than being surface-induced; the formation of another surface between mannitol crystals and the amorphous phase would exacerbate the loss of protein recovery.

In the case of LDH, the mechanism of denaturation is mainly ascribable to surface adsorption onto ice.^25^ As can be seen from Figure 2c, a slow freeze/thawing protocol preserved the LDH activity more than fast freezing in liquid nitrogen; the faster the freezing was, the larger the ice/freeze-concentrate surface area was and, hence, the surface-induced denaturation of LDH. The denaturing action of TBA on LDH was even more pronounced than for Mb. The recovery of LDH was about 35% for all the formulations containing TBA, independently of the excipients added. In the absence of TBA, sucrose preserved LDH activity more than mannitol, and the cyclodextrin was the best excipient among those tested in this work. The addition of Tween 80 to the TBA-free formulations was also highly effective at preventing LDH denaturation. The surfactant could locate at interfaces, displacing LDH from the surface and effectively promoting its enzymatic activity recovery. This result confirms once again the surface-driven nature of the LDH denaturation. A similar effect of Tween 80 was not observed for Mb, whose denaturation is not ascribable to its surface adsorption. On the contrary, Tween 80 may even have a denaturing action on Mb, boosting its cold unfolding, as observed in a previous work.^17^

### TBA Denatures Myoglobin in Molecular Dynamics Simulations

The results reported in this section refer to simulations performed with both the TBAff descriptions, as detailed previously in the Methods. Any difference between the results obtained from the two parameterizations has been pointed out. Further details regarding the validation of the parameterizations are reported in the corresponding section of the Supporting Information.

The analysis of the average radius of gyration (Table S2, see Supporting Information) suggested that, even if the two TBAff descriptions provided quantitatively different results, they both agreed on the following aspect: TBA and the A/W interface had a destabilizing effect on myoglobin compactness. In particular, the addition of TBA systematically caused partial unfolding, while the A/W interface had a milder impact on the Mb compactness. Furthermore, the overall destabilizing action was reduced whenever TBA was present in combination with the A/W interface. Lastly, the hydrophobic fraction of the Mb solvent-accessible surface area increased in the presence of TBA (Table S3, see Supporting Information), and this was primarily observed in the aqueous bulk. The addition of TBA provoked a shift of the surface hydrophobicity towards higher values, while the presence of the A/W interface did not seem to cause major changes.

**Figure 3:**
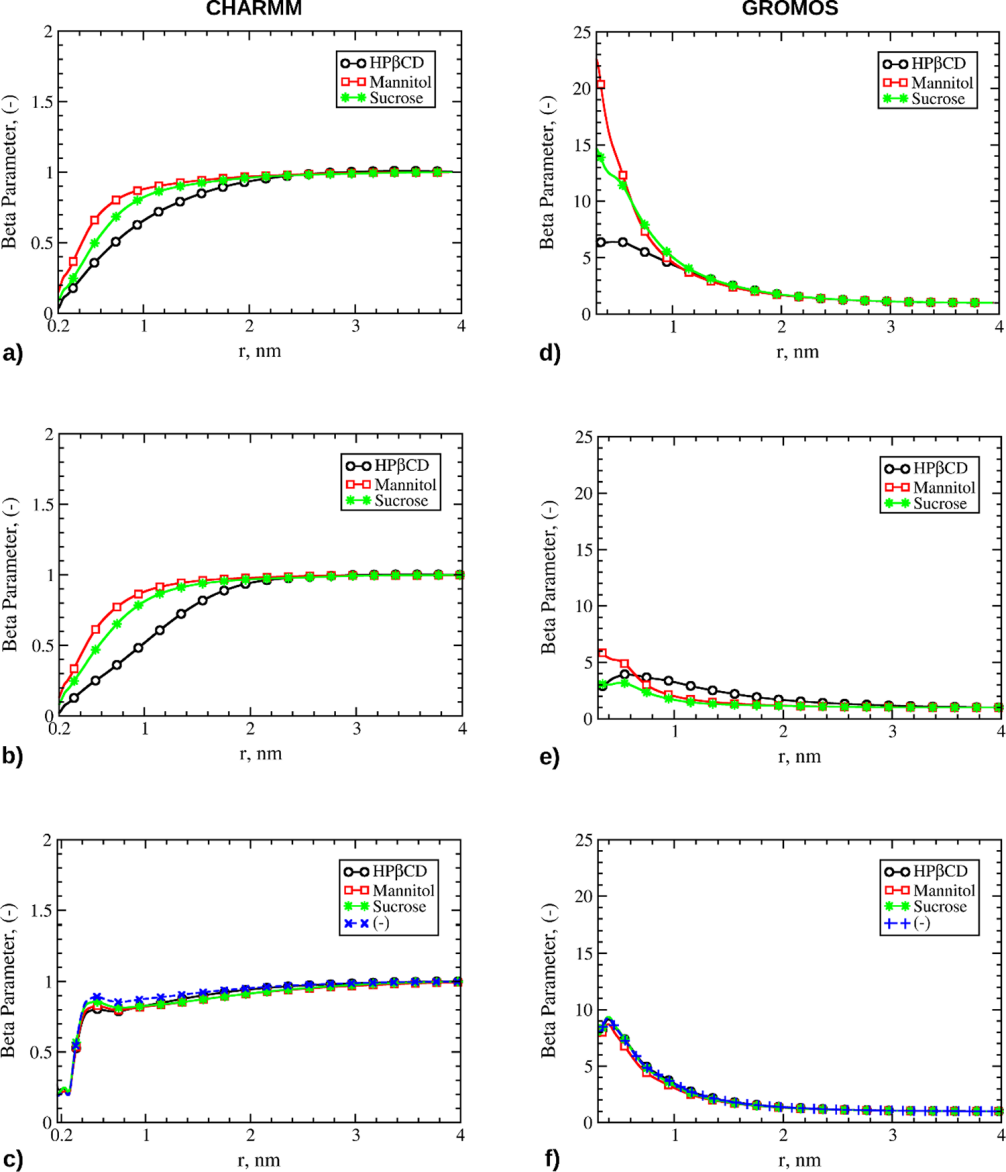
β-Parameter profiles for the various formulations containing Mb in the liquid bulk, corresponding to simulations from 2b to 3d of Table 3. Left column: Mb parameterized with the CHARMM36m force field. Right column: Mb parameterized with the GROMOS 54a7 force field. The legend box indicates the excipients in solution, if any. Panels a and d: profiles for the excipients in bulk water, simulations 2b–2d. Panels b and e: profiles for the excipients in the 20% w/w TBA bulk solution, simulations 3b–3d. Panels c and f: profiles for TBA in the 20% w/w TBA bulk solution, simulations 3a-3d.

The β-parameter profiles obtained from the two TBAff parameterizations were significantly different in quantitative terms, although they provided similar qualitative information to understand the role of excipients and TBA in the different formulations. For example, the CHARMM36m description of the protein showed preferential exclusion from the Mb surface for all solutes in the aqueous bulk, namely excipients and TBA (Figure 3a–c). However, the β-parameter profiles for TBA showed a bump at a distance of approximately 0.3 nm from the peptide surface (Figure 3c), which indicates the presence of a thin shell of TBA molecules in close proximity to the protein, as represented in Figure S9. Analogous behavior was observed at the A/W interface, as shown in Figure S6a–c. On the other hand, the GROMOS description of the protein suggested a strong preferential inclusion of the excipients in the aqueous bulk (Figure 3d).

Figure 3e shows that the addition of TBA reduced the value of the β-parameter, thus the amount of excipient molecules near the Mb surface, as the cosolvent molecules were more included. The simulations carried out using the GROMOS parameterization showed that the TBA molecules accumulated around Mb (Figure 3f), thereby promoting the exclusion of the excipients. Similar behavior was observed at the A/W interface (Figure S6d–f). Nevertheless, the addition of TBA made the excipients more preferentially included at the A/W interface with respect to the corresponding bulk systems. At the same time, the TBA accumulation was slightly reduced at the A/W interface with respect to the bulk systems.

Independently of the adopted force field description, TBA has been shown to accumulate closely around the Mb surface. For this reason, we decided to further analyze the interactions between TBA and the peptide residues. Although the fraction of the overall time spent by the TBA molecules near the Mb surface varied with the formulation composition, TBA exhibited a clear tendency to accumulate nearby positively charged peptide residues (Figure S10, see Supporting Information), especially in close proximity of lysine. Although the two force field descriptions resulted in significantly different quantitative estimations of the time spent by TBA molecules near the peptide residues, both models confirmed this behavior.

Dimensionless density profiles confirmed some of the previous results. As shown in Figure S7a,d, the excipients density at the A/W interface was not null, although significant accumulation at the interface was only observed for HPβCD. Similar results were previously observed in the literature.^57^ Instead, when TBA molecules were added (sims. 5b–5d), they accumulated at the A/W interface (Figure S7c,f), thus the excipients were confined in the bulk of the system (Figure S7b,e). It follows that the reduced impact of TBA on the Mb compactness (Table S2) at the A/W interface may be explained by the distinct accumulation of TBA near the gaseous phase, reducing the amount of TBA molecules available in the liquid bulk to interact with the protein itself. Mb mainly accumulated in the liquid bulk in sims. 4a–4d (Figure S8a,c); however, ambiguous results were observed in the presence of TBA (sims. 5a–5d), as can be seen in Figure S8b,d. The CHARMM36m description predicted that Mb remained in the liquid bulk (Figure S8b), whereas the GROMOS (sim. 5a) showed the preferential accumulation of the protein at the A/W interface (Figure S8d). In fact, Mb has been shown to be susceptible to denaturation at gaseous/liquid interfaces and, thus, to potentially undergo aggregation.^63^ The addition of excipients appreciably reduced the amount of protein at the interface, although only the mannitol formulation (sim. 5c) seemed to be able to completely prevent interfacial adsorption, according to both force field descriptions (Figure S8b,d). This result may be explained by the β-parameter profiles, as mannitol was more preferentially included than TBA around Mb close to the A/W interface, while the opposite behavior was observed for all the other excipients investigated (see Figure S6b,d,e,f).

The computational results are in agreement with the experimental evidences of TBA-induced denaturation previously discussed. It may be concluded that the pronounced reduction of the Mb recovery would be caused by the accumulation of the TBA molecules nearby the protein surface, decreasing its structural compactness and promoting the solvation of otherwise buried hydrophobic residues. Similarly, the experimentally observed inability of excipients to provide a satisfactory stabilizing action (Figure 2) can be explained using the β-parameter profiles collected through MD simulations. According to such profiles, the excipients cannot displace TBA from the peptide surface. Mb prevalently interacts with the TBA molecules, thus eliminating any kind of stabilizing action by the excipients. These results agreed with what previously observed by Bellezza et al.,^13^ who observed that Mb formulations containing TBA experienced a reduction of the thermal stability with increasing TBA concentration. Specifically, they found that Mb exhibited a decreased enzymatic activity with increasing TBA content, which was then found to be related to the hydrophobic clustering^13^ of TBA molecules. This clustering phenomenon was here confirmed by the *G*_TT_ plots shown in Figure S3a (which represent TBA-TBA interactions in terms of KBI, see Supporting Information for further details), with molar fractions ranging from 0.04 to 0.1 (corresponding to mass fractions roughly within 0.146 and 0.314, range that includes the 0.20 value used for the simulations). The slope of the curve is positive and the sign changes from negative to positive, thus suggesting favorable TBA–TBA interactions. Bellezza et al.^13^ suggested that cosolvents could solvate proteins by displacing water molecules from their surfaces, in agreement with the β-parameter profiles here presented. However, Bellezza et al.^13^ hypothesized that increasing concentrations of TBA would favor interactions between the cosolvent and non-polar residues, whereas the molecular simulations here revealed that, despite an increase in the hydrophobicity of the Mb surface, the TBA molecules preferentially interact with positively charged residues (see Figure S10).

## Conclusions

The TBA-water formulations of biopharmaceutical products represent one possible solution to ensure protein stability.^9^ However, the interplay between TBA, excipients and process conditions is often complex, thus making the formulation design and development a challenging task. For this reason, two model proteins, namely lactate dehydrogenase and myoglobin, were used in this work as case studies to investigate the extent of surface-induced and cold denaturation in cosolvent formulations.

Initially, the formulations were thermally characterized by means of DSC and FDM, then the recovery of protein activity was evaluated after either freezing or freeze-drying. While the tested excipients (mannitol, sucrose and HPβCD) could provide a stabilizing action compared to the corresponding buffer-only formulation, the recovery of activity was drastically reduced when TBA was added. TBA has been shown to be responsible for a dramatic loss of protein recovery, and the tested excipients were not able to counteract the negative impact of the cosolvent.

Molecular dynamics was then used to better understand the nature of the TBA destabilizing effect. Simulations revealed that TBA accumulated in proximity of the Mb surface, especially near positively charged residues, while pushing away the excipients from the peptide surface. The addition of TBA increased both the radius of gyration and the fraction of hydrophobic solvent accessible surface area of Mb, i.e., a decreased structural compactness probably driven by the exposure of previously buried non-polar residues. Furthermore, simulations revealed that TBA accumulated at the air–water interface. This behavior reduced the negative impact of TBA on the Mb structural stability, as a smaller number of TBA molecules were available in the liquid bulk, therefore limiting their detrimental interaction with the protein.

Although TBA speeds up the freeze-drying process^9^ and, consequently, reduces the time spent in potentially denaturing conditions, its addition to either LDH or Mb formulations led to a marked loss of activity. It could be suggested that the non-polar character of TBA may cause an effect similar to cold denaturation,^64^ namely favoring the solvation of otherwise inaccessible hydrophobic residues.^13^ It might be possible that other non-polar cosolvents could cause a similar effect on proteins, although further research would be needed in this direction.

MD simulations provided valuable information at the molecular scale, otherwise unattainable by experiments. The combined experimental-computational approach here proposed may serve as a guideline for future research, especially to investigate and isolate the causes, at a molecular level, of destabilizing phenomena induced by complex mixture of organic solvents.

## Supporting Information Available

XRD analysis of mannitol’s polymorphic states; Development of a TBA Force Field**;** Myoglobin setup; RMSD time profiles for myoglobin’s formulations; Radius of gyration values for myoglobin’s simulations; Solvent accessible surface area values for myoglobin’s simulations; β-parameter profiles for myoglobin’s formulations at the air–water interface; Density profiles for myoglobin’s formulations at the air–water interface; Representation of TBA accumulation around myoglobin’s surface (PDF)

## Abbreviations

MD: molecular dynamics
TBA: *tert*-butanol
LDH: lactate dehydrogenase
Mb: myoglobin
HPβCD: 2-hydroxypropyl-β-cyclodextrin
DSC: differential scanning calorimetry
FDM: freeze-drying microscopy
XRD: X-ray diffractometry
OD: optical density
ANOVA: analysis of variance
HSD: honestly significant difference
KBI(s): Kirkwood-Buff integral(s)
sim(s): simulation(s)
comb. rule: combination rule
A/W: air–water
*T*′_g_: glass transition temperature
*T*_cr_: crystallization temperature
*T*_eu_: eutectic melting temperature
*T*_c_: collapse temperature

## References

[ref1] Moorkens E.; Meuwissen N.; Huys I.; Declerck P.; Vulto A. G.; Simoens S. The Market of Biopharmaceutical Medicines: A Snapshot of a Diverse Industrial Landscape. Front. Pharmacol. 2017, 8, 314 10.3389/fphar.2017.00314. 28642701 PMC5462923

[ref2] Franks F.; Auffret T. Freeze-Drying of Pharmaceuticals and Biopharmaceuticals; The Royal Society of Chemistry, 2007.

[ref3] Wang W. Lyophilization and Development of Solid Protein Pharmaceuticals. Int. J. Pharm. 2000, 203, 1–60. 10.1016/S0378-5173(00)00423-3. 10967427

[ref4] Wang W.; Roberts C. J. Aggregation of Therapeutic Proteins, 2010.

[ref5] Moussa E. M.; Panchal J. P.; Moorthy B. S.; Blum J. S.; Joubert M. K.; Narhi L. O.; Topp E. M. Immunogenicity of Therapeutic Protein Aggregates. J. Pharm. Sci. 2016, 105, 417–430. 10.1016/j.xphs.2015.11.002. 26869409

[ref6] Kolář P.; Shen J.-W.; Tsuboi A.; Ishikawa T. Solvent Selection for Pharmaceuticals. Fluid Phase Equilib. 2002, 194–197, 771–782. 10.1016/S0378-3812(01)00716-6.

[ref7] Teagarden D. L.; Baker D. S. Practical Aspects of Lyophilization Using Non-Aqueous Co-Solvent Systems. Eur. J. Pharm. Sci. 2002, 15, 115–133. 10.1016/S0928-0987(01)00221-4. 11849908

[ref8] Vessot S.; Andrieu J. A Review on Freeze Drying of Drugs with Tert-Butanol (TBA) + Water Systems: Characteristics, Advantages, Drawbacks. Drying Technol. 2012, 30, 377–385. 10.1080/07373937.2011.628133.

[ref9] Teagarden D.L.; Wang W.; Baker D.S. Practical aspects of freeze-drying of pharmaceutical and biological products using nonaqueous cosolvent systems. In Freeze Drying/Lyophilization of Pharmaceutical and Biological Products, 3rd ed.; Rey L.; May J. C., Eds.; Informa Healthcare: London, UK, 2010; pp 254–287.

[ref10] Pisano R.; Fissore D.; Barresi A. A. Noninvasive Monitoring of a Freeze-Drying Process for Tert-Butanol/Water Cosolvent-Based Formulations. Ind. Eng. Chem. Res. 2016, 55, 5670–5680. 10.1021/acs.iecr.5b04299.

[ref11] Kasraian K.; DeLuca P. P. The Effect of Tertiary Butyl Alcohol on the Resistance of the Dry Product Layer During Primary Drying. Pharm. Res. 1995, 12, 491–495. 10.1023/A:1016285425670. 7596982

[ref12] Wang Z.; Deng Y.; Zhang X. The Novel Application of Tertiary Butyl Alcohol in the Preparation of Hydrophobic Drug-HPβCD Complex. J. Pharm. Pharmacol. 2006, 58, 409–414. 10.1211/jpp.58.3.0017. 16536910

[ref13] Bellezza F.; Cipiciani A.; Cinelli S.; Onori G. Influence of Alcohols and Osmolytes on Thermal Stability and Catalytic Activity of Myoglobin: Co-Solvent Clustering Effects. Chem. Phys. Lett. 2009, 482, 139–142. 10.1016/j.cplett.2009.09.103.

[ref14] Magsumov T.; Ziying L.; Sedov I. Comparative Study of the Protein Denaturing Ability of Different Organic Cosolvents. Int. J. Biol. Macromol. 2020, 160, 880–888. 10.1016/j.ijbiomac.2020.05.260. 32497668

[ref15] Yong Z.; Yingjie D.; Xueli W.; Jinghua X.; Zhengqiang L. Conformational and Bioactivity Analysis of Insulin: Freeze-Drying TBA/Water Co-Solvent System in the Presence of Surfactant and Sugar. Int. J. Pharm. 2009, 371, 71–81. 10.1016/j.ijpharm.2008.12.018. 19136051

[ref16] Sonje J.; Thakral S.; Suryanarayanan R. T-Butanol Enables Dual Functionality of Mannitol: A Cryoprotectant in Frozen Systems and Bulking Agent in Freeze-Dried Formulations. Mol. Pharmaceutics 2020, 17, 3075–3086. 10.1021/acs.molpharmaceut.0c00492. 32633520

[ref17] Arsiccio A.; Giorsello P.; Marenco L.; Pisano R. Considerations on Protein Stability During Freezing and Its Impact on the Freeze-Drying Cycle: A Design Space Approach. J. Pharm. Sci. 2020, 109, 464 10.1016/j.xphs.2019.10.022. 31647953

[ref18] Arsiccio A.; Marenco L.; Pisano R. A Model-Based Approach for the Rational Design of the Freeze-Thawing of a Protein-Based Formulation. Pharm. Dev. Technol. 2020, 25, 823–831. 10.1080/10837450.2020.1743719. 32367756

[ref19] Attig N. Computational Soft Matter: From Synthetic Polymers to Proteins: Lecture Notes, John von Neumann-Institut Für Computing. In NIC series; NIC, 2004; Vol. 23.

[ref20] Arsiccio A.; Paladini A.; Pattarino F.; Pisano R. Designing the Optimal Formulation for Biopharmaceuticals: A New Approach Combining Molecular Dynamics and Experiments. J. Pharm. Sci. 2019, 108, 431–438. 10.1016/j.xphs.2018.09.002. 30222976

[ref21] Arsiccio A.; Pisano R. Design of the Formulation for Therapeutic Proteins How to Improve Stability of Drugs during Freezing and in the Dried State. Chim. Oggi/Chem. Today 2018, 36, 16–18.

[ref22] Arsiccio A.; Pisano R. Water Entrapment and Structure Ordering as Protection Mechanisms for Protein Structural Preservation. J. Chem. Phys. 2018, 148, 55102 10.1063/1.5012884. 29421888

[ref23] Humphrey W.; Dalke A.; Schulten K. VMD: Visual Molecular Dynamics. J. Mol. Graphics 1996, 14, 33–38. 10.1016/0263-7855(96)00018-5. 8744570

[ref24] Chang B. S.; Randall C. S. Use of Subambient Thermal Analysis to Optimize Protein Lyophilization. Cryobiology 1992, 29, 632–656. 10.1016/0011-2240(92)90067-C.

[ref25] Bhatnagar B. S.; Pikal M. J.; Bogner R. H. Study of the Individual Contributions of Ice Formation and Freeze-Concentration on Isothermal Stability of Lactate Dehydrogenase during Freezing. J. Pharm. Sci. 2008, 97, 798–814. 10.1002/jps.21017. 17506511

[ref26] Patel S. M.; Doen T.; Pikal M. J. Determination of End Point of Primary Drying in Freeze-Drying Process Control. AAPS PharmSciTech 2010, 11, 73–84. 10.1208/s12249-009-9362-7. 20058107 PMC2850457

[ref27] Pisano R. Automatic Control of a Freeze-Drying Process: Detection of the End Point of Primary Drying. Drying Technol. 2020, 40, 140–157. 10.1080/07373937.2020.1774891.

[ref28] Anderson A. B.; Robertson C. R. Absorption Spectra Indicate Conformational Alteration of Myoglobin Adsorbed on Polydimethylsiloxane. Biophys. J. 1995, 68, 2091–2097. 10.1016/S0006-3495(95)80388-7. 7612852 PMC1282113

[ref29] Abraham M. J.; Murtola T.; Schulz R.; Páll S.; Smith J. C.; Hess B.; Lindah E. Gromacs: High Performance Molecular Simulations through Multi-Level Parallelism from Laptops to Supercomputers. SoftwareX 2015, 1–2, 19–25. 10.1016/j.softx.2015.06.001.

[ref30] Oostenbrink C.; Villa A.; Mark A. E.; Van Gunsteren W. F. A Biomolecular Force Field Based on the Free Enthalpy of Hydration and Solvation: The GROMOS Force-Field Parameter Sets 53A5 and 53A6. J. Comput. Chem. 2004, 25, 1656–1676. 10.1002/jcc.20090. 15264259

[ref31] Pol-Fachin L.; Rusu V. H.; Verli H.; Lins R. D. GROMOS 53A6GLYC, an Improved GROMOS Force Field for Hexopyranose-Based Carbohydrates. J. Chem. Theory Comput. 2012, 8, 4681–4690. 10.1021/ct300479h. 26605624

[ref32] Lee M. E.; van der Vegt N. F. A. A New Force Field for Atomistic Simulations of Aqueous Tertiary Butanol Solutions. J. Chem. Phys. 2005, 122, 114509 10.1063/1.1862625. 15836231

[ref33] Kirkwood J. G.; Buff F. P. The Statistical Mechanical Theory of Solutions. I. J. Chem. Phys. 1951, 19, 774–777. 10.1063/1.1748352.

[ref34] Nishikawa K.; Kodera Y.; Iijima T. Fluctuations in the Particle Number and Concentration and the Kirkwood-Buff Parameters of Tert-Butyl Alcohol and Water Mixtures Studied by Small-Angle X-Ray Scattering. J. Phys. Chem. 1987, 91, 3694–3699. 10.1021/j100297a047.

[ref35] Berendsen H. J. C.; Grigera J. R.; Straatsma T. P. The Missing Term in Effective Pair Potentials. J. Phys. Chem. 1987, 91, 6269–6271. 10.1021/j100308a038.

[ref36] MacKerell A. D. Jr.; Bashford D.; Bellott M.; Dunbrack R. L. Jr.; Evanseck J. D.; Field M. J.; Fischer S.; Gao J.; Guo H.; Ha S.; Joseph-McCarthy D.; Kuchnir L.; Kuczera K.; Lau F. T. K.; Mattos C.; Michnick S.; Ngo T.; Nguyen D. T.; Prodhom B.; Reiher W. E. III; Roux B.; Schlenkrich M.; Smith J. C.; Stote R.; Straub J.; Watanabe M.; Wiórkiewicz-Kuczera J.; Yin D.; Karplus M. All-Atom Empirical Potential for Molecular Modeling and Dynamics Studies of Proteins. J. Phys. Chem. B 1998, 102, 3586–3616. 10.1021/jp973084f. 24889800

[ref37] Berendsen H. J. C.; Postma J. P. M.; Van Gunsteren W. F.; Dinola A.; Haak J. R. Molecular Dynamics with Coupling to an External Bath. J. Chem. Phys. 1984, 81, 3684–3690. 10.1063/1.448118.

[ref38] Essmann U.; Perera L.; Berkowitz M. L.; Darden T.; Lee H.; Pedersen L. G. A Smooth Particle Mesh Ewald Method. J. Chem. Phys. 1995, 103, 8577–8593. 10.1063/1.470117.

[ref39] Hess B.; Bekker H.; Berendsen H. J. C.; Fraaije J. G. E. M. LINCS: A Linear Constraint Solver for Molecular Simulations. J. Comput. Chem. 1997, 18, 1463–1472. 10.1002/(SICI)1096-987X(199709)18:12<1463::AID-JCC4>3.0.CO;2-H.

[ref40] Miyamoto S.; Kollman P. A. Settle: An Analytical Version of the SHAKE and RATTLE Algorithm for Rigid Water Models. J. Comput. Chem. 1992, 13, 952–962. 10.1002/jcc.540130805.

[ref41] Nosé S. A Unified Formulation of the Constant Temperature Molecular Dynamics Methods. J. Chem. Phys. 1984, 81, 511–519. 10.1063/1.447334.

[ref42] Nosé S. A Molecular Dynamics Method for Simulations in the Canonical Ensemble. Mol. Phys. 2002, 100, 191–198. 10.1080/00268970110089108.

[ref43] Hoover W. G. Canonical Dynamics: Equilibrium Phase-Space Distributions. Phys. Rev. A 1985, 31, 1695–1697. 10.1103/PhysRevA.31.1695. 9895674

[ref44] Parrinello M.; Rahman A. Polymorphic Transitions in Single Crystals: A New Molecular Dynamics Method. J. Appl. Phys. 1981, 52, 7182–7190. 10.1063/1.328693.

[ref45] Ganguly P.; Van Der Vegt N. F. A. Convergence of Sampling Kirkwood-Buff Integrals of Aqueous Solutions with Molecular Dynamics Simulations. J. Chem. Theory Comput. 2013, 9, 1347–1355. 10.1021/ct301017q. 26587597

[ref46] Berman H. M.; Westbrook J.; Feng Z.; Gilliland G.; Bhat T. N.; Weissig H.; Shindyalov I. N.; Bourne P. E. The Protein Data Bank/Biopython. Nucleic Acids Res. 2000, 28, 235–242. 10.1093/nar/28.1.235. 10592235 PMC102472

[ref47] Maurus R.; Overall C. M.; Bogumil R.; Luo Y.; Mauk A. G.; Smith M.; Brayer G. D. A Myoglobin Variant with a Polar Substitution in a Conserved Hydrophobic Cluster in the Heme Binding Pocket. Biochim. Biophys. Acta, Protein Struct. Mol. Enzymol. 1997, 1341, 1–13. 10.1016/S0167-4838(97)00064-2. 9300804

[ref48] Anandakrishnan R.; Aguilar B.; Onufriev A. V. H++ 3.0: Automating PK Prediction and the Preparation of Biomolecular Structures for Atomistic Molecular Modeling and Simulations. Nucleic Acids Res. 2012, 40, W537–W541. 10.1093/nar/gks375. 22570416 PMC3394296

[ref49] Bussi G.; Donadio D.; Parrinello M. Canonical Sampling through Velocity Rescaling. J. Chem. Phys. 2007, 126, 14101 10.1063/1.2408420. 17212484

[ref50] Huang J.; Rauscher S.; Nawrocki G.; Ran T.; Feig M.; De Groot B. L.; Grubmüller H.; MacKerell A. D. Jr. CHARMM36m: An Improved Force Field for Folded and Intrinsically Disordered Proteins. Nat. Methods 2017, 14, 71–73. 10.1038/nmeth.4067. 27819658 PMC5199616

[ref51] Schmid N.; Eichenberger A. P.; Choutko A.; Riniker S.; Winger M.; Mark A. E.; Van Gunsteren W. F. Definition and Testing of the GROMOS Force-Field Versions 54A7 and 54B7. Eur. Biophys. J. 2011, 40, 843–856. 10.1007/s00249-011-0700-9. 21533652

[ref52] Lin Z.; Van Gunsteren W. F. Refinement of the Application of the GROMOS 54A7 Force Field to β-Peptides. J. Comput. Chem. 2013, 34, 2796–2805. 10.1002/jcc.23459. 24122968

[ref53] Poger D.; Van Gunsteren W. F.; Mark A. E. A New Force Field for Simulating Phosphatidylcholine Bilayers. J. Comput. Chem. 2010, 31, 1117–1125. 10.1002/jcc.21396. 19827145

[ref54] Arsiccio A.; Ganguly P.; La Cortiglia L.; Shea J.-E.; Pisano R. The ADD Force Field for Sugars and Polyols: Predicting the Additivity of Protein-Osmolyte Interaction. J. Phys. Chem. B 2020, 124, 7779–7790. 10.1021/acs.jpcb.0c05345. 32790371 PMC7901642

[ref55] Arsiccio A.; Rospiccio M.; Shea J.-E.; Pisano R. Force Field Parameterization for the Description of the Interactions between Hydroxypropyl-β-Cyclodextrin and Proteins. J. Phys. Chem. B 2021, 125, 7397–7405. 10.1021/acs.jpcb.1c04033. 34210121 PMC8287564

[ref56] Eisenhaber F.; Lijnzaad P.; Argos P.; Sander C.; Scharf M. The Double Cubic Lattice Method: Efficient Approaches to Numerical Integration of Surface Area and Volume and to Dot Surface Contouring of Molecular Assemblies. J. Comput. Chem. 1995, 16, 273–284. 10.1002/jcc.540160303.

[ref57] Rospiccio M.; Arsiccio A.; Winter G.; Pisano R. The Role of Cyclodextrins against Interface-Induced Denaturation in Pharmaceutical Formulations: A Molecular Dynamics Approach. Mol. Pharmaceutics 2021, 18, 2322–2333. 10.1021/acs.molpharmaceut.1c00135. PMC8289300 33999634

[ref58] Meredith P.; Donald A. M.; Payne R. S. Freeze-Drying: In Situ Observations Using Cryoenvironmental Scanning Electron Microscopy and Differential Scanning Calorimetry. J. Pharm. Sci. 1996, 85, 631–637. 10.1021/js950324z. 8773961

[ref59] Kasraian K.; Spitznagel T. M.; Juneau J. A.; Yim K. Characterization of the Sucrose/Glycine/Water System by Differential Scanning Calorimetry and Freeze-Drying Microscopy. Pharm. Dev. Technol. 1998, 3, 233–239. 10.3109/10837459809028500. 9653761

[ref60] Bhatnagar B. S.; Sonje J.; Shalaev E.; Martin S. W. H.; Teagarden D. L.; Suryanarayanan R. A Refined Phase Diagram of the: *tert*-Butanol-Water System and Implications on Lyophilization Process Optimization of Pharmaceuticals. Phys. Chem. Chem. Phys. 2020, 22, 1583–1590. 10.1039/c9cp06576h. 31894786

[ref61] Kasraian K.; DeLuca P. P. Thermal Analysis of the Tertiary Butyl Alcohol-Water System and Its Implications on Freeze-Drying. Pharm. Res. 1995, 12, 484–490. 10.1023/A:1016233408831. 7596981

[ref62] Thakral S.; Sonje J.; Munjal B.; Bhatnagar B.; Suryanarayanan R. Mannitol as an Excipient for Lyophilized Injectable Formulations. J. Pharm. Sci. 2023, 112, 19–35. 10.1016/j.xphs.2022.08.029. 36030846

[ref63] Xiao Y.; Konermann L. Protein Structural Dynamics at the Gas/Water Interface Examined by Hydrogen Exchange Mass Spectrometry. Protein Sci. 2015, 24, 1247–1256. 10.1002/pro.2680. 25761782 PMC4534175

[ref64] Dias C. L.; Ala-Nissila T.; Wong-ekkabut J.; Vattulainen I.; Grant M.; Karttunen M. The Hydrophobic Effect and Its Role in Cold Denaturation. Cryobiology 2010, 60, 91–99. 10.1016/j.cryobiol.2009.07.005. 19616532

