# Supplementary material for: Mechanistic Investigation of tert-Butanol’s Impact on Biopharmaceutical Formulations: When Experiments Meet Molecular Dynamics
Source: Mol Pharm. 2023 Jul 12;20(8):3975–86. doi: 10.1021/acs.molpharmaceut.3c00125 (PMC10410665; doi:10.1021/acs.molpharmaceut.3c00125)
Supplement: Supplementary file 1 — mp3c00125_si_001.pdf [file mp3c00125_si_001.pdf]

Supporting Information for:

Mechanistic Investigation of *tert*-Butanol's Impact on Biopharmaceutical Formulations: When Experiments Meet Molecular Dynamics

*Marcello Rospiccio, Paola Casucci, Andrea Arsiccio, Claudia Udrescu & Roberto Pisano**

Molecular Engineering Laboratory, Department of Applied Science and Technology, Politecnico di Torino, Torino 10129, Italy.

*Corresponding author: roberto.pisano@polito.it

Abbreviations: XRD, X-ray diffractometry; TBA, *tert*-butanol; HPβCD, 2-hydroxypropyl-β-cyclodextrin; Mb, myoglobin; A/W, air-water; KBI(s), Kirkwood-Buff integral(s), Sim(s), simulation(s); comb. rule, combination rule; RMSD, root mean square deviation; std. dev., standard deviation.

XRD analysis of mannitol's polymorphic states

The crystallization of mannitol is influenced by the manufacturing conditions and has been discussed in the literature[1–3]. In fact, the various mannitol anhydrous polymorphs (α, β, and δ) show different thermodynamic stability, which can have an impact on the final features of the active pharmaceutical ingredients. The thermodynamically stable form of mannitol is the β polymorph, while the α and δ polymorphs are metastable[1]. Thus, controlling the crystallization of mannitol is fundamental to guarantee the stability of lyophilized drugs. For this reason, we investigated the effect of TBA and Tween 80 on mannitol polymorphs' formation. Table S1 shows the results of the XRD characterization for the lyophilized mannitol formulations, as listed in Table 1, produced following the freeze-drying protocol described in Table 2 (see manuscript), although without any proteins. Formation of δ-mannitol with traces of α- and β- for Mo and Mo* formulations was observed, while for formulations M and M* both δ- and β- forms were detected. The corresponding XRD patterns are reported in Figures S1 and S2. The addition of TBA to mannitol formulations promoted the formation of the δ-form, in agreement with reports in literature[4,5].

Table S1. Mannitol polymorphs observed in the lyophilized products.

Code	Formulation	Mannitol polymorphs
M	5% w/w Mannitol	δ and β
M*	5% w/w Mannitol + 0.01% w/v Tween 80	δ and β
Mo	5% w/w Mannitol + 20% w/w TBA	δ with traces of α and β
Mo*	5% w/w Mannitol + 20% w/w TBA + 0.01% w/v Tween 80	δ with traces of α and β

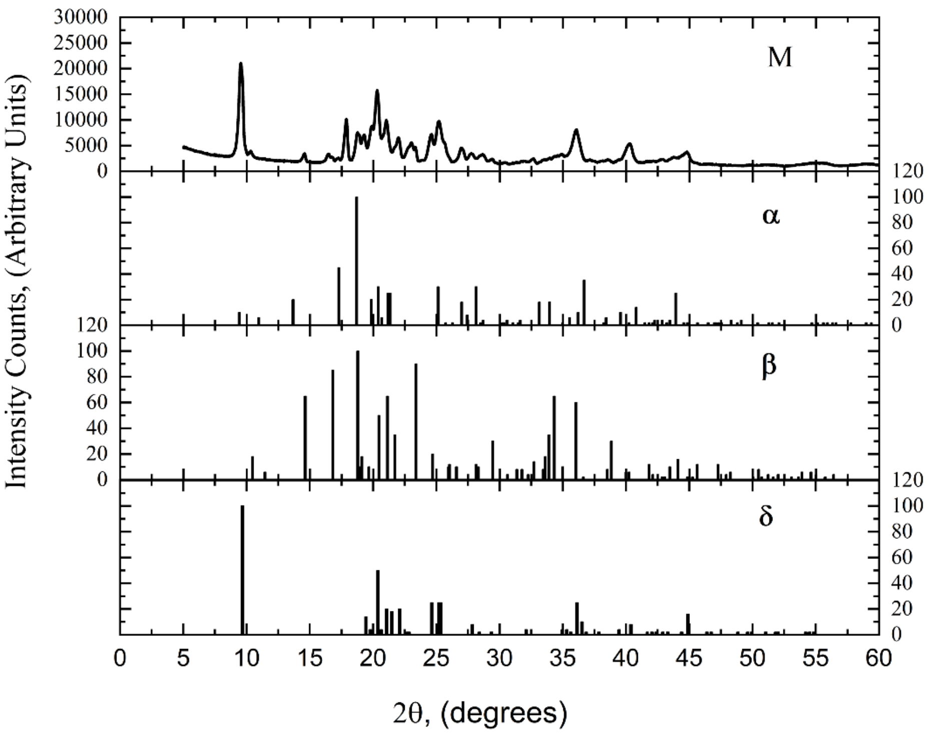

Figure S1. XRD pattern of freeze-dried mannitol (M, as indicated in Table 1 of the manuscript) and mannitol's anhydrous polymorphs reference spectra (α, β and δ).

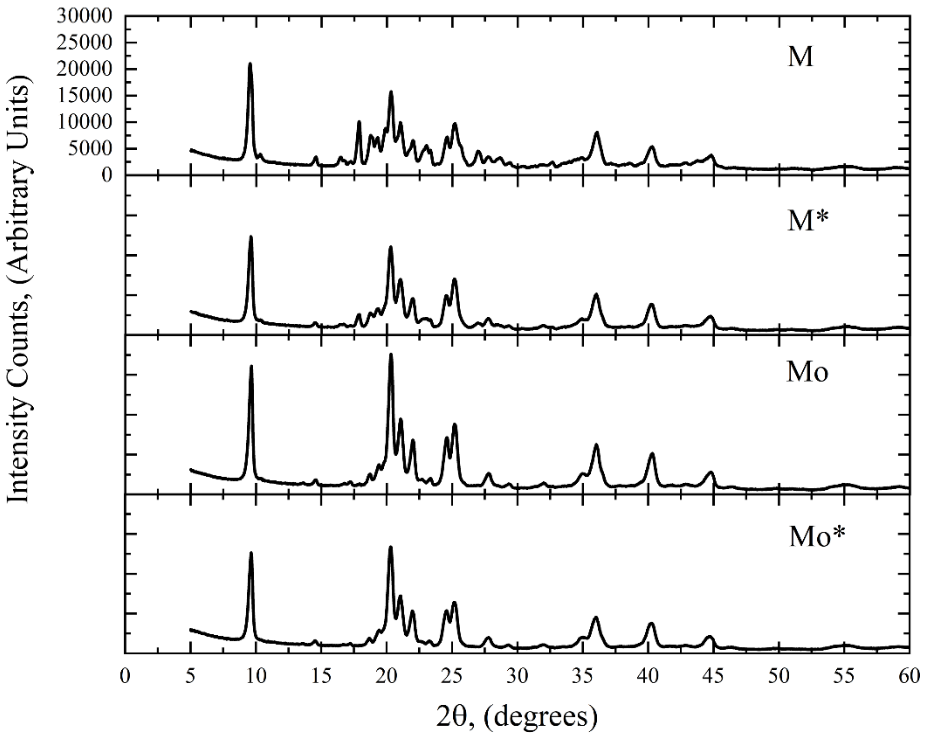

Figure S2. XRD pattern of freeze-dried mannitol formulations as indicated in Table 1 of the manuscript.

Development of a TBA Force Field

The Kirkwood Buff Integrals[6] (KBIs, see Eq. 1 of the manuscript) computed for both the original and modified van der Vegt parameterizations of TBA (sims. type 1 of Table 3, see manuscript) are shown in Figure S3. Although Di Pierro et al.[7] improved the Lee and van der Vegt[8] description of TBA-TBA interactions, which are expressed as the TBA-TBA Kirkwood-Buff Integral G_{TT} (Figure S3a), their model cannot reproduce the negative sign of the experimental G_{TT} at the lowest concentration. For this reason, we here implemented a modified parameterization for the nonbonded interactions of TBA's oxygen atom with its carbon atoms, named TBAff. We observed that the TBAff description accurately predicted the TBA-TBA interactions in the case of SPCE[9]/combination rule 1 parameters, whereas the CHARMM TIP3P[10]/combination rule 2 description was inaccurate at low concentrations, which are, unfortunately, the range of interest of this study. Similarly, Figure S3b shows the TBA-water interactions, evaluated in terms of the TBA-water Kirkwood-Buff integral G_{TW}, which were accurately described by the SPCE/combination rule 1 but rather poorly by the CHARMM TIP3P/combination rule 2 parameterization. Instead, Figure S3c shows that the two TBAff descriptions for the water-water interactions, evaluated in terms of the water-water Kirkwood-Buff integral G_{WW}, show fluctuations.

Overall, the SPCE/combination rule 1 parametrization approximated the experimental KBIs quite well, while the CHARMM TIP3P/combination rule 2 parameterization was inaccurate at low concentrations. The simulations of the same systems with larger boxes (8 nm edge) confirmed the results observed for the smaller systems. Despite the shortcomings of the CHARMM TIP3P/combination rule 2 parameterization, we presented its results as they could still provide valuable qualitative information concerning the TBA behavior and a term for comparison.

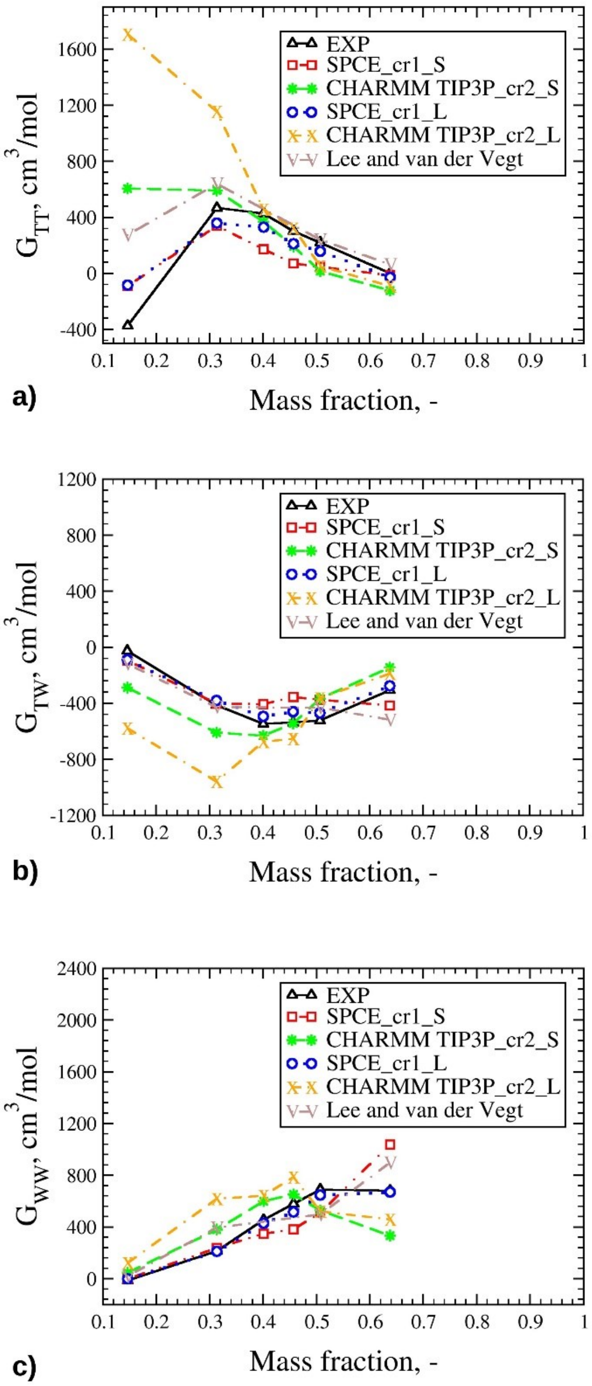

Figure S3. KBIs for the TBA-water mixtures corresponding to simulations 1a-f (see Table 3 of the manuscript). G_{TT}, G_{TW}, and G_{WW} refer to TBA-TBA, TBA-water, and water-water interactions, respectively. The 'EXP' label refers to experimental data from Nishikawa et al.[11], 'Lee and van der Vegt' indicates the original Lee and van der Vegt description[8], while the 'CHARMM TIP3P_cr2' and 'SPCE_cr1' labels indicate the data obtained from our modified van der Vegt parameterizations. The labels 'S' and 'L' refer to the systems of size 4x4x4 nm and 8x8x8 nm, respectively.

Myoglobin setup

The molecular structure of myoglobin was obtained from the RCSB PDB data bank[12], PDB code 1WLA[13]. The protein conformation at pH 3.7 was then obtained from the H++ server[14], resulting in a charge equal to +10, which was balanced by the addition of a corresponding number of Cl^- counterions. Mb was described using both the CHARMM36m[15] force field in combination with CHARMM TIP3P water and the GROMOS 54a7[16–18] force field with SPCE water. To assess if the resulting structures were equilibrated, a simulation in water was performed, as detailed in the corresponding section of the manuscript (Simulation of Myoglobin Formulations).

The time profiles of RMSD (root mean square deviation) were evaluated for both topologies. The RMSD profiles for Mb's peptide backbone were computed with the *gmx rms* built-in Gromacs command. Specifically, the reference structure was the structure resulting from the removal of the heteroatoms and the charge adjustment to reproduce the conformation at pH 3.7. Such profiles are reported in Figure S4. Thus, it could be observed that the structures were equilibrated throughout the simulation, as the RMSD fluctuations over time were limited after the initial 20 ns.

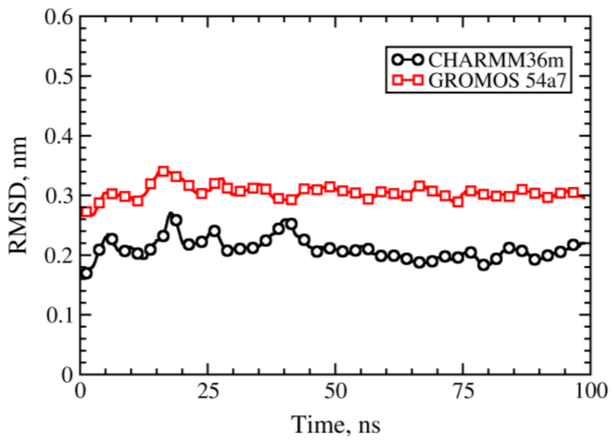

Figure S4. RMSD time profiles for myoglobin's peptide backbone comparing the two force field descriptions, namely CHARMM36m force field in combination with CHARMM TIP3P water and the GROMOS 54a7 force field with SPCE water.

RMSD time profiles for myoglobin's formulations

To verify that the simulation setup was adequate to observe the onset of any unfolding/denaturation behavior, as well as long enough to obtain equilibrated configurations, the RMSD time profiles were computed for the peptide backbone of Mb. To do so, firstly the simulations of Mb in bulk water without excipients were used to extract the reference structure for the computation of the RMSD profiles. Specifically, the *gmx cluster* built-in Gromacs command was used, using the Daura algorithm[19]. The mean structure of the prevalent structure cluster was extracted and hence used as reference. The procedure was performed for both force field descriptions of Mb. It was then possible to compute the RMSD time profiles with such structures as reference. An example of such profiles is reported in Figure S5, where the RMSD time profiles were reported for systems containing Mb without excipients (corresponding to sims. 2a, 3a, 4a, 5a of Table 3 of the manuscript).

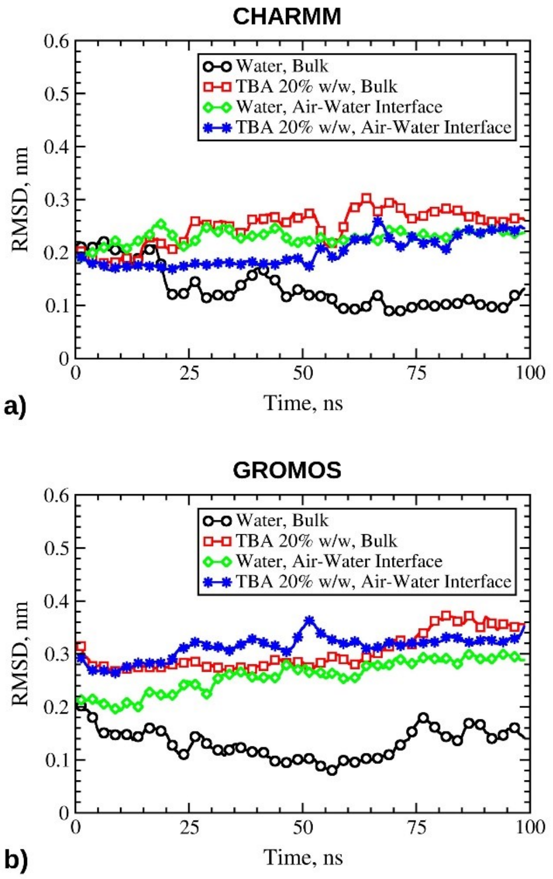

Figure S5. RMSD time profiles for Mb's peptide backbone comparing the two force field descriptions, namely CHARMM36m force field in combination with CHARMM TIP3P water (panel a) and the GROMOS 54a7

force field with SPCE water (panel b). The four cases reported in the legend box correspond to the sims. 2a, 3a, 4a, 5a of Table 3 of the manuscript.

Overall, the trend of the RMSD values is in agreement with the radius of gyration ones, presented in the following section and discussed in the manuscript (see the 'TBA Denatures Myoglobin in Molecular Dynamics Simulations' section). Thus, it could be concluded that the onset of denaturation was observed throughout the simulations, as the values of RMSD were higher whenever TBA and/or interfaces were present, and that the observed conformational state of Mb was equilibrated.

Radius of gyration values for myoglobin's simulations

In this section, the time-averaged values of the radius of gyration for all myoglobin's simulations are reported. The time average was computed over the last 80 ns of the simulations.

Table S2. Time-averaged values of the radius of gyration of myoglobin for all systems, and associated standard deviation (std. dev.).

	**Average Radius of Gyration (std. dev.), nm**			
	**Aqueous Bulk**		**Air-Water Interface**	
	**0% w/w TBA**	**20% w/w TBA**	**0% w/w TBA**	**20% w/w TBA**
	**CHARMM36m**			
**Myoglobin**	1.550 (0.011)	1.612 (0.018)	1.563 (0.012)	1.581 (0.021)
**HPβCD**	1.543 (0.012)	1.583 (0.013)	1.548 (0.010)	1.576 (0.012)
**Mannitol**	1.564 (0.016)	1.573 (0.013)	1.544 (0.009)	1.588 (0.013)
**Sucrose**	1.552 (0.011)	1.618 (0.027)	1.559 (0.012)	1.595 (0.033)
	**GROMOS 54a7**			
**Myoglobin**	1.533 (0.010)	1.599 (0.019)	1.541 (0.017)	1.598 (0.012)
**HPβCD**	1.539 (0.010)	1.626 (0.013)	1.545 (0.010)	1.648 (0.013)
**Mannitol**	1.526 (0.009)	1.568 (0.007)	1.536 (0.009)	1.587 (0.017)
**Sucrose**	1.520 (0.006)	1.582 (0.010)	1.560 (0.009)	1.573 (0.008)

Solvent accessible surface area values for myoglobin's simulations

In this section, the time-averaged values of the hydrophobic fraction of solvent accessible surface area for all myoglobin's simulations are reported. The time average was computed over the last 80 ns of the simulations.

Table S3. Time-averaged values of the hydrophobic fraction of myoglobin's solvent accessible surface area for all systems, and associated standard deviation (std. dev.).

	**Fraction of Hydrophobic Solvent Accessible Surface (std. dev.), %**			
	**Aqueous Bulk**		**Air-Water Interface**	
	**0% w/w TBA**	**20% w/w TBA**	**0% w/w TBA**	**20% w/w TBA**
	**CHARMM36m**			
**Myoglobin**	49.88 (1.09)	52.79 (1.11)	50.42 (0.93)	51.69 (1.09)
**HPβCD**	50.61 (1.06)	52.00 (1.22)	50.05 (0.97)	52.15 (1.08)
**Mannitol**	50.67 (1.15)	52.86 (1.06)	49.02 (0.99)	52.07 (1.03)
**Sucrose**	50.21 (1.16)	53.01 (1.13)	50.96 (1.19)	52.94 (1.58)
	**GROMOS 54a7**			
**Myoglobin**	42.71 (1.08)	44.92 (0.97)	43.32 (1.04)	45.74 (1.07)
**HPβCD**	42.39 (1.01)	47.07 (0.83)	42.07 (1.00)	47.49 (1.11)
**Mannitol**	41.16 (0.78)	43.96 (0.77)	40.57 (0.80)	45.19 (0.96)
**Sucrose**	41.62 (1.00)	46.08 (1.08)	41.67 (0.85)	45.44 (0.89)

β-parameter profiles for myoglobin's formulations at the air-water interface

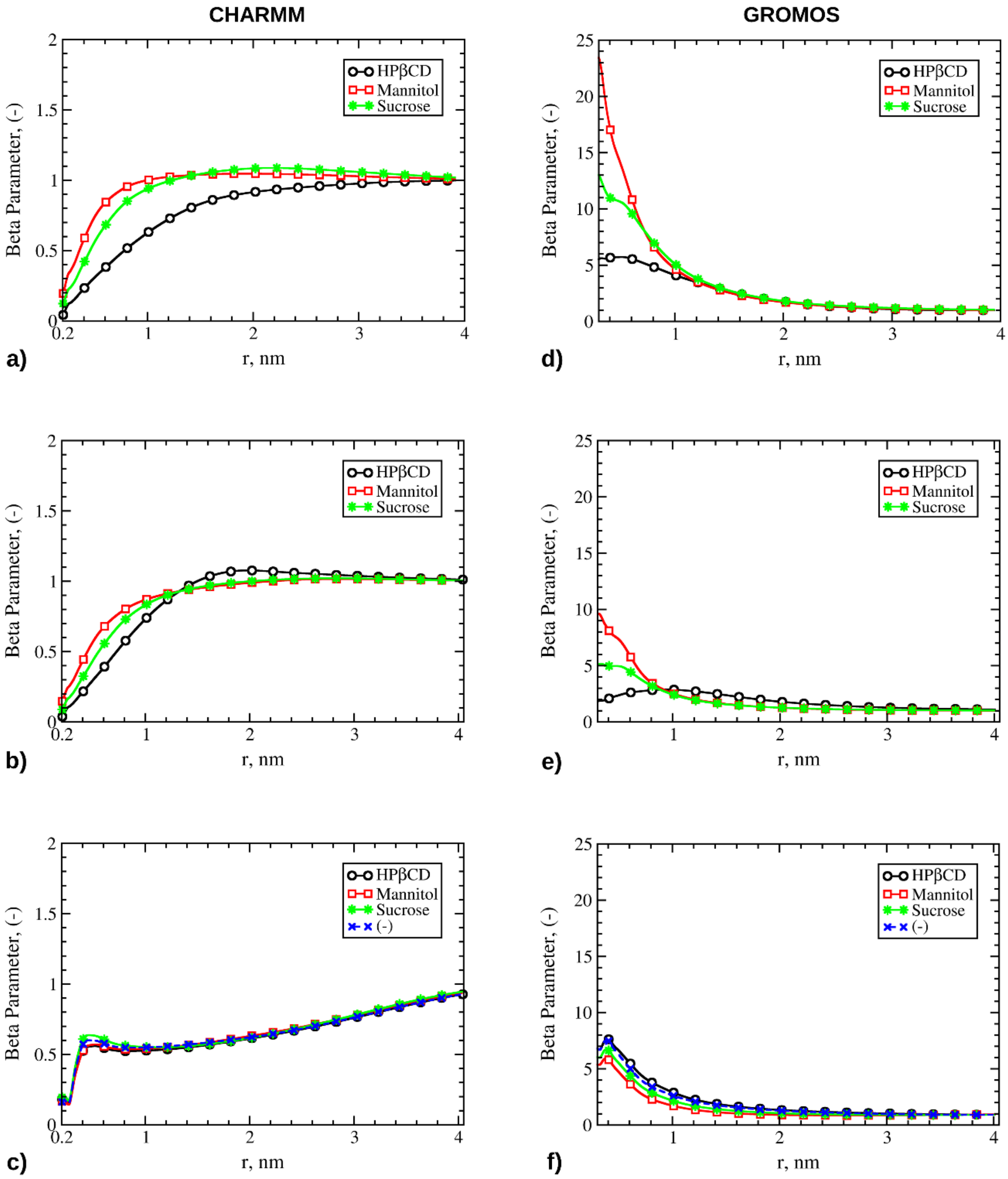

Figure S6. β-parameter profiles for the various formulations containing Mb at the air-water interface, corresponding to simulations from 4b to 5d of Table 3 of the manuscript. Left column: Mb parameterized with the CHARMM36m force field. Right column: Mb parameterized with the GROMOS 54a7 force field. The legend box indicates the excipients in solution, if any. Panels a and d: profiles for the excipients at the A/W interface, simulations 4b-4d. Panels b and e: profiles for the excipients at the A/W interface, with 20% w/w

TBA in solution, simulations 5b-5d. Panels c and f: profiles for TBA at the A/W interface, with 20% w/w TBA in solution, simulations 5a-5d.

Density profiles for myoglobin's formulations at the air-water interface

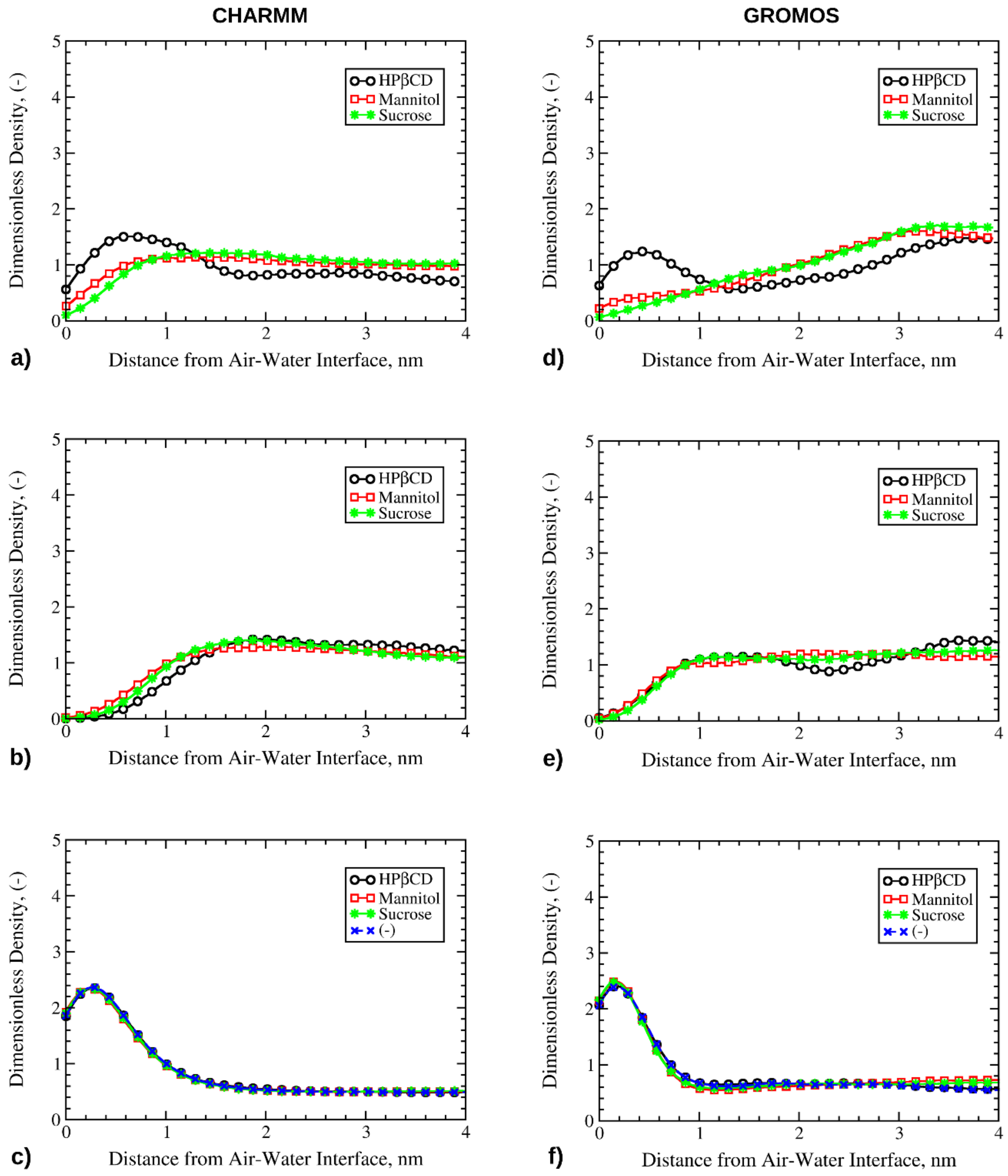

Figure S7. Dimensionless density profiles for excipients and TBA at the A/W interface, corresponding to simulations from 4b to 5d of Table 3 of the manuscript. Left column: Mb parameterized with the CHARMM36m force field. Right column: Mb parameterized with the GROMOS 54a7 force field. The legend box indicates the excipients in solution, if any. Panels a and d: profiles for the excipients at the A/W interface, simulations 4b-4d. Panels b and e: profiles for the excipients at the A/W interface, with 20% w/w TBA in solution, simulations 5b-5d. Panels c and f: profiles for TBA at the A/W interface, with 20% w/w TBA in solution, simulations 5a-5d.

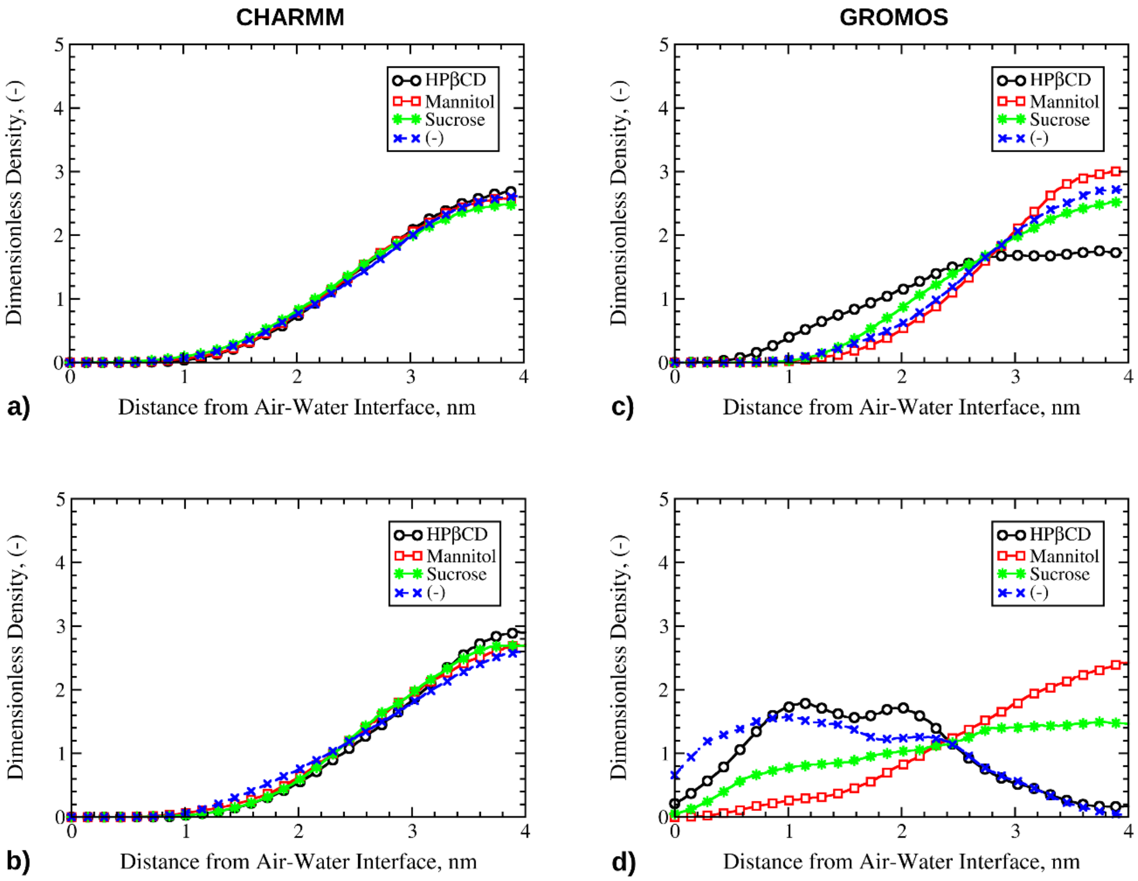

Figure S8. Dimensionless density profiles of myoglobin at the A/W interface, corresponding to simulations from 4a to 5d of Table 3 of the manuscript. Left column: Mb parameterized with the CHARMM36m force field. Right column: Mb parameterized with the GROMOS 54a7 force field. The legend box indicates the excipients in solution, if any. Panels a and c: profiles for myoglobin at the A/W interface, simulations 4a-4d. Panels b and d: profiles for myoglobin at the air-water interface, with 20% w/w TBA, simulations 5a-5d.

Accumulation of TBA molecules around myoglobin's surface

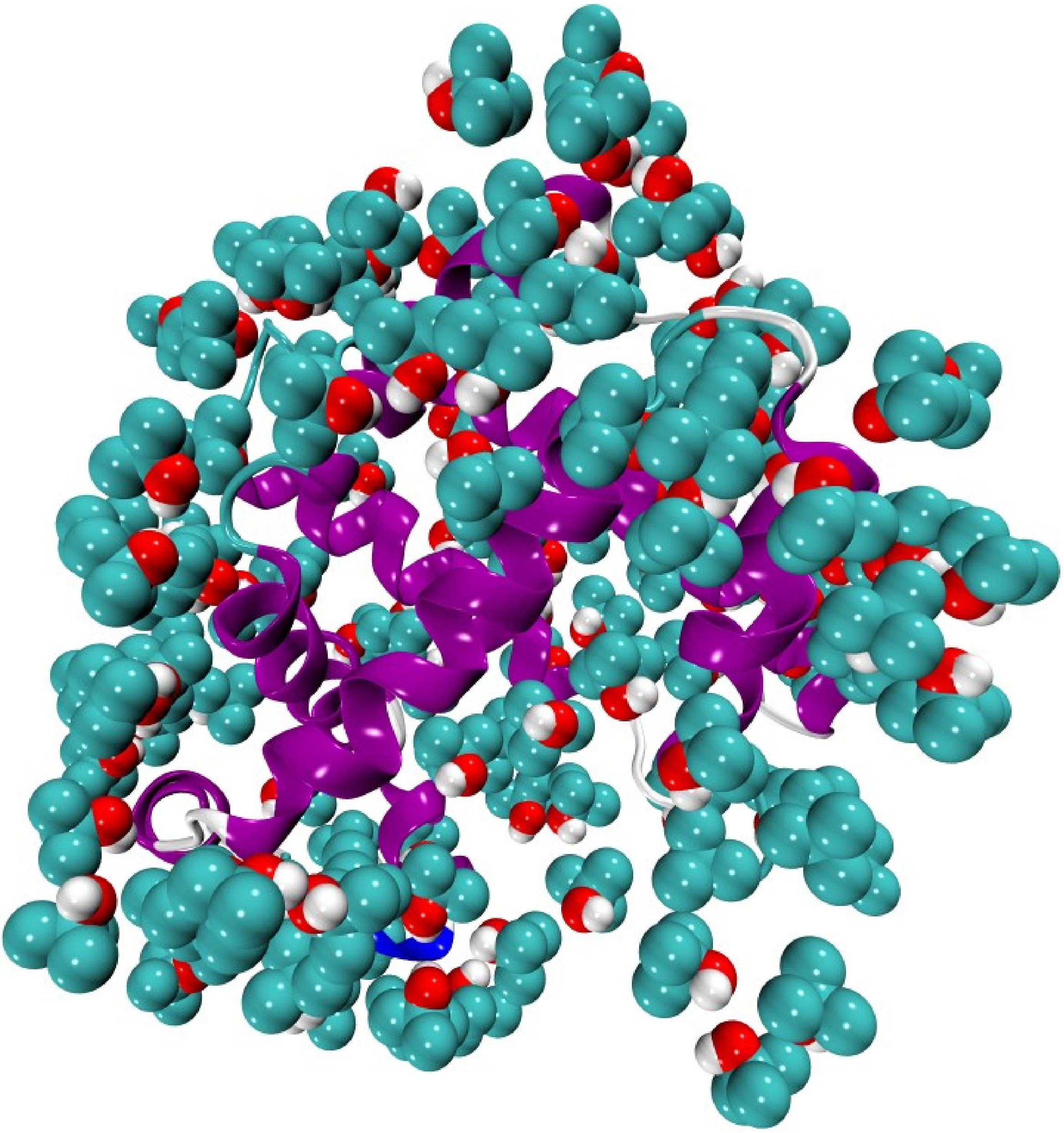

Figure S9. Representation of TBA molecules accumulating in proximity of myoglobin's surface. Snapshot realized with Visual Molecular Dynamics 1.9.3 (VMD)[20].

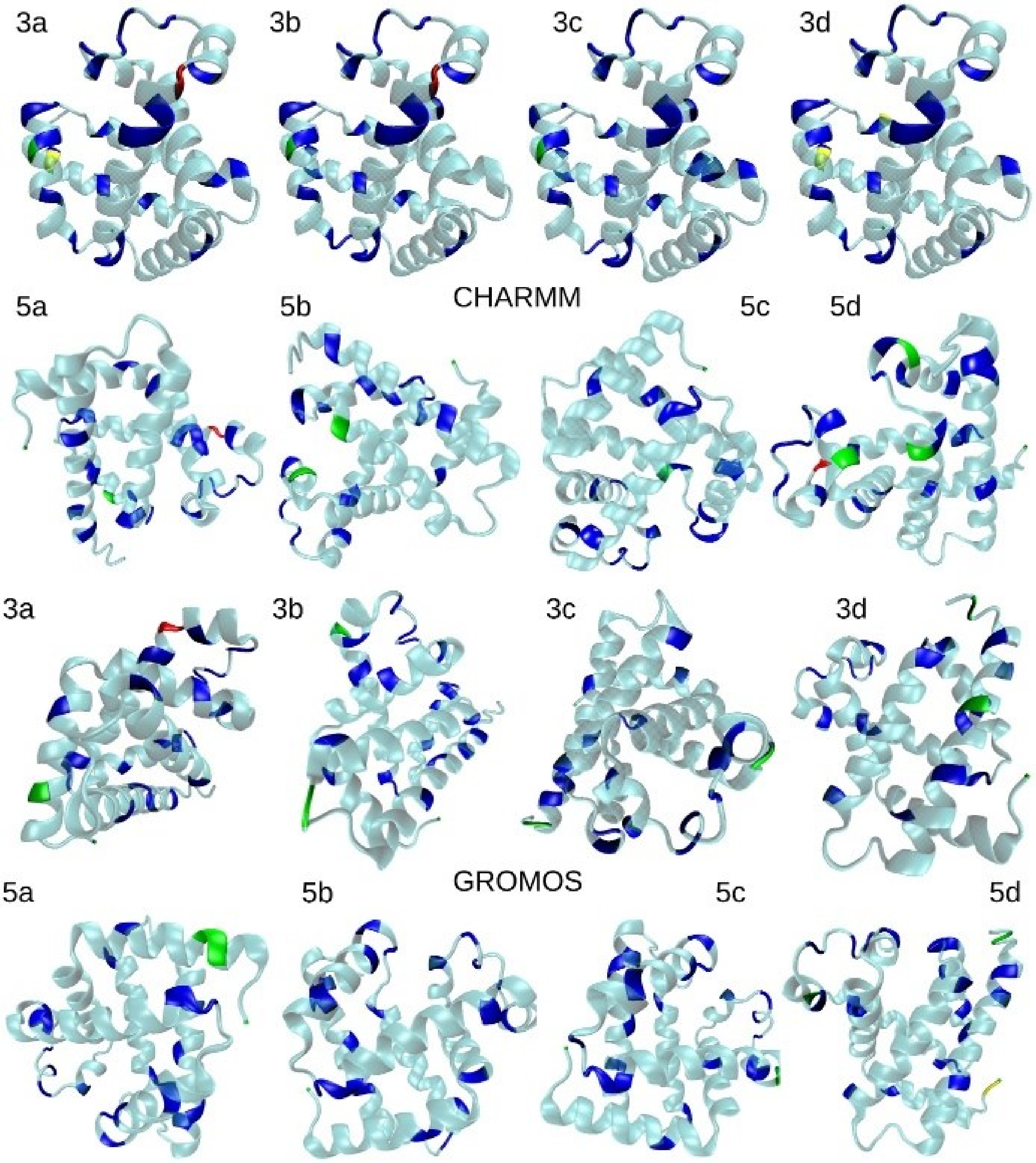

Figure S10. Representation of the peptide residues around which TBA molecules accumulate. Color code: blue, positively charged residues; red, negatively charged residues; green, polar residues; yellow, non-polar residues. The labels refer to the corresponding type of simulation (see Table 3 of the manuscript). The first two rows refer to results obtained with the CHARMM36m[15] description of myoglobin, while the last two rows refer to the GROMOS 54a7[16–18] description. Snapshots were realized with Visual Molecular Dynamics 1.9.3 (VMD)[20].

AUTHOR INFORMATION

Corresponding Author

*Email: roberto.pisano@polito.it

Notes

The authors declare no competing financial interest.

ACKNOWLEDGMENTS

Computational resources were provided by HPC@POLITO, a project of Academic Computing within the Department of Control and Computer Engineering at the Politecnico di Torino (https://hpc.polito.it), and by CINECA under the ISCRA initiative (CD-AA-FF-HP10C1LPRF and EXCINCOS-HP10CPG3PV). The Authors wish to thank Joanny Drevillon and Julia Amici for their contribution to the experimental campaign.

REFERENCES

(1) Liao, X.; Krishnamurthy, R.; Suryanarayanan, R. Influence of Processing Conditions on the Physical State of Mannitol - Implications in Freeze-Drying. *Pharm. Res.* **2007**, *24* (2), 370 – 376. https://doi.org/10.1007/s11095-006-9158-3.

(2) Yang, Y.; Liu, J.; Hu, A.; Nie, T.; Cheng, Z.; Liu, W. A Critical Review on Engineering of D-Mannitol Crystals: Properties, Applications, and Polymorphic Control. *Crystals* **2022**, *12* (8). https://doi.org/10.3390/cryst12081080.

(3) Kim, A. I.; Akers, M. J.; Nail, S. L. The Physical State of Mannitol after Freeze-Drying: Effects of Mannitol Concentration, Freezing Rate, and a Noncrystallizing Cosolute. *J. Pharm. Sci.* **1998**, *87* (8), 931–935. https://doi.org/10.1021/js980001d.

(4) Sonje, J.; Thakral, S.; Suryanarayanan, R. T-Butanol Enables Dual Functionality of Mannitol:

A Cryoprotectant in Frozen Systems and Bulking Agent in Freeze-Dried Formulations. *Mol. Pharm.* **2020**, *17* (8), 3075–3086. https://doi.org/10.1021/acs.molpharmaceut.0c00492.

(5) Thakral, S.; Sonje, J.; Munjal, B.; Bhatnagar, B.; Suryanarayanan, R. Mannitol as an Excipient for Lyophilized Injectable Formulations. *J. Pharm. Sci.* **2023**, *112* (1), 19–35. https://doi.org/10.1016/j.xphs.2022.08.029.

(6) Kirkwood, J. G.; Buff, F. P. The Statistical Mechanical Theory of Solutions. I. *J. Chem. Phys.* **1951**, *19* (6), 774–777. https://doi.org/10.1063/1.1748352.

(7) Di Pierro, M.; Mugnai, M. L.; Elber, R. Optimizing Potentials for a Liquid Mixture: A New Force Field for a Tert-Butanol and Water Solution. *J. Phys. Chem. B* **2015**, *119* (3), 836–849. https://doi.org/10.1021/jp505401m.

(8) Lee, M. E.; van der Vegt, N. F. A. A New Force Field for Atomistic Simulations of Aqueous Tertiary Butanol Solutions. *J. Chem. Phys.* **2005**, *122* (11), 114509. https://doi.org/10.1063/1.1862625.

(9) Berendsen, H. J. C.; Grigera, J. R.; Straatsma, T. P. The Missing Term in Effective Pair Potentials. *J. Phys. Chem.* **1987**, *91* (24), 6269–6271. https://doi.org/10.1021/j100308a038.

(10) MacKerell Jr., A. D.; Bashford, D.; Bellott, M.; Dunbrack Jr., R. L.; Evanseck, J. D.; Field, M. J.; Fischer, S.; Gao, J.; Guo, H.; Ha, S.; Joseph-McCarthy, D.; Kuchnir, L.; Kuczera, K.; Lau, F. T. K.; Mattos, C.; Michnick, S.; Ngo, T.; Nguyen, D. T.; Prodhom, B.; Reiher III, W. E.; Roux, B.; Schlenkrich, M.; Smith, J. C.; Stote, R.; Straub, J.; Watanabe, M.; Wiórkiewicz-Kuczera, J.; Yin, D.; Karplus, M. All-Atom Empirical Potential for Molecular Modeling and Dynamics Studies of Proteins. *J. Phys. Chem. B* **1998**, *102* (18), 3586–3616. https://doi.org/10.1021/jp973084f.

(11) Nishikawa, K.; Kodera, Y.; Iijima, T. Fluctuations in the Particle Number and Concentration

and the Kirkwood-Buff Parameters of Tert-Butyl Alcohol and Water Mixtures Studied by Small-Angle X-Ray Scattering. *J. Phys. Chem.* **1987**, *91* (13), 3694–3699. https://doi.org/10.1021/j100297a047.

(12) Berman, H. M. The Protein Data Bank / Biopython. *Presentation* **2000**, *28* (1), 235–242. https://doi.org/10.1093/nar/28.1.235.

(13) Maurus, R.; Overall, C. M.; Bogumil, R.; Luo, Y.; Mauk, A. G.; Smith, M.; Brayer, G. D. A Myoglobin Variant with a Polar Substitution in a Conserved Hydrophobic Cluster in the Heme Binding Pocket. *Biochim. Biophys. Acta - Protein Struct. Mol. Enzymol.* **1997**, *1341* (1), 1–13. https://doi.org/10.1016/S0167-4838(97)00064-2.

(14) Anandakrishnan, R.; Aguilar, B.; Onufriev, A. V. H++ 3.0: Automating PK Prediction and the Preparation of Biomolecular Structures for Atomistic Molecular Modeling and Simulations. *Nucleic Acids Res.* **2012**, *40* (W1), W537–W541. https://doi.org/10.1093/nar/gks375.

(15) Huang, J.; Rauscher, S.; Nawrocki, G.; Ran, T.; Feig, M.; De Groot, B. L.; Grubmüller, H.; MacKerell A.D., J. CHARMM36m: An Improved Force Field for Folded and Intrinsically Disordered Proteins. *Nat. Methods* **2016**, *14* (1), 71–73. https://doi.org/10.1038/nmeth.4067.

(16) Schmid, N.; Eichenberger, A. P.; Choutko, A.; Riniker, S.; Winger, M.; Mark, A. E.; Van Gunsteren, W. F. Definition and Testing of the GROMOS Force-Field Versions 54A7 and 54B7. *Eur. Biophys. J.* **2011**, *40* (7), 843–856. https://doi.org/10.1007/s00249-011-0700-9.

(17) Lin, Z.; Van Gunsteren, W. F. Refinement of the Application of the GROMOS 54A7 Force Field to β-Peptides. *J. Comput. Chem.* **2013**, *34* (32), 2796–2805. https://doi.org/10.1002/jcc.23459.

(18) Poger, D.; Van Gunsteren, W. F.; Mark, A. E. A New Force Field for Simulating Phosphatidylcholine Bilayers. *J. Comput. Chem.* **2010**, *31* (6), 1117–1125.

https://doi.org/10.1002/jcc.21396.

(19) Daura, X.; Gademann, K.; Jaun, B.; Seebach, D.; van Gunsteren, W. F.; Mark, A. E. Peptide Folding: When Simulation Meets Experiment. *Angew. Chemie Int. Ed.* **1999**, *38* (1-2), 236–240. https://doi.org/10.1002/(SICI)1521-3773(19990115)38:1/2<236::AID-ANIE236>3.0.CO;2-M.

(20) Humphrey, W.; Dalke, A.; Schulten, K. VMD: Visual Molecular Dynamics. *J. Mol. Graph.* **1996**, *14* (1), 33–38. https://doi.org/10.1016/0263-7855(96)00018-5.
